# A high‐throughput FTIR spectroscopy approach to assess adaptive variation in the chemical composition of pollen

**DOI:** 10.1002/ece3.3619

**Published:** 2017-11-09

**Authors:** Boris Zimmermann, Murat Bağcıoğlu, Valeria Tafinstseva, Achim Kohler, Mikael Ohlson, Siri Fjellheim

**Affiliations:** ^1^ Faculty of Life Science and Technology Norwegian University of Life Sciences Ås Norway; ^2^ Nofima AS Ås Norway; ^3^ Faculty of Environmental Sciences and Natural Resource Management Norwegian University of Life Sciences Ås Norway; ^4^ Faculty of Biosciences Norwegian University of Life Sciences Ås Norway

**Keywords:** adaptation, *Anthoxanthum*, ecology and environmental sciences, *Festuca*, flowering, Fourier transform infrared spectroscopy, grasses, *Poa*, Poaceae, sparse partial least‐squares regression

## Abstract

The two factors defining male reproductive success in plants are pollen quantity and quality, but our knowledge about the importance of pollen quality is limited due to methodological constraints. Pollen quality in terms of chemical composition may be either genetically fixed for high performance independent of environmental conditions, or it may be plastic to maximize reproductive output under different environmental conditions. In this study, we validated a new approach for studying the role of chemical composition of pollen in adaptation to local climate. The approach is based on high‐throughput Fourier infrared (FTIR) characterization and biochemical interpretation of pollen chemical composition in response to environmental conditions. The study covered three grass species, *Poa alpina*,* Anthoxanthum odoratum*, and *Festuca ovina*. For each species, plants were grown from seeds of three populations with wide geographic and climate variation. Each individual plant was divided into four genetically identical clones which were grown in different controlled environments (high and low levels of temperature and nutrients). In total, 389 samples were measured using a high‐throughput FTIR spectrometer. The biochemical fingerprints of pollen were species and population specific, and plastic in response to different environmental conditions. The response was most pronounced for temperature, influencing the levels of proteins, lipids, and carbohydrates in pollen of all species. Furthermore, there is considerable variation in plasticity of the chemical composition of pollen among species and populations. The use of high‐throughput FTIR spectroscopy provides fast, cheap, and simple assessment of the chemical composition of pollen. In combination with controlled‐condition growth experiments and multivariate analyses, FTIR spectroscopy opens up for studies of the adaptive role of pollen that until now has been difficult with available methodology. The approach can easily be extended to other species and environmental conditions and has the potential to significantly increase our understanding of plant male function.

## 1 INTRODUCTION

Pollen and ovules are the two key players in one of the most fundamental of all processes in plant biology—fertilization. While the female function is well described, the biology of the male function is less investigated. The difference in weight given to male and female reproductive output in research can be ascribed to the relative ease with which the female reproductive output is measured compared to that of pollen. However, given its fundamental role in reproduction, we expect that the male function is under strong selective pressure to maximize reproductive success.

Pollen quantity and quality are key determinants of male reproductive success that both can limit fertilization through quantity in terms of pollen availability and through quality in terms of pollen viability, germination capacity, and tube development rate (Arceo‐Gomez et al., 2016; Williams & Mazer, 2016). We may expect trade‐offs between quantity and distribution on one side and viability, germination, growth, and fertilization success on the other side. Two main drivers of pollen quality evolution are widely accepted: One is pollination mode (by wind, water, or animals), and the other is the length the pollen tube needs to grow from the stigma to the ovule, that is, that pollen of species with long distances needs to be rich in energy and nutrients (Lau & Stephenson, 1993). Consequently, the chemical composition of pollen varies between species and families (Baker & Baker, 1979) and may be an important component of pollen quality. Insect pollinated species, for example, frequently use pollen as reward for pollinators and have in general higher protein and/or oil content (Jones & Little, 1983). Accordingly, Hanley, Franco, Pichon, Darvill, and Goulson (2008) have shown that plants with high pollen protein content attract more insects. In comparison, wind‐ or self‐pollinated species such as grasses have high starch contents (Speranza, Calzoni, & Pacini, 1997; Wang, Ge, Scott, & Spangenberg, 2004). The evolutionary rationale is that the metabolic cost of producing proteins and oils are higher than carbohydrates, and plants with nonrewarding pollen (e.g., wind pollinators, self‐pollinators, and nectar rewarders) will thus be characterized by pollen that are more rich in carbohydrates.

Given its fundamental importance, the chemical composition of pollen may be genetically fixed for high performance independent of environmental conditions. Alternatively, it may be plastic to maximize reproductive output under different environmental conditions. One of the most studied and influential factors limiting pollen growth and development is temperature (Hedhly, Hormaza, & Herrera, 2004; Rang, Jagadish, Zhou, Craufurd, & Heuer, 2011), but the studies are mainly focused on the conditions during germination and pollen tube growth (Alcaraz, Montserrat, & Hormaza, 2011; Pham, Herrero, & Hormaza, 2015). Although several studies have provided examples of how environmental conditions during pollen maturation influence their chemical composition (Lau & Stephenson, 1994; Stanley & Linskens, 1974; Zimmermann & Kohler, 2014), the role of pollen chemical composition in adaptation is rarely investigated.

Standard pollen analysis is predominantly obtained by optical microscopy in order to provide information on pollen morphology and germination (Kakani et al., 2005; Koti, Reddy, Kakani, Zhao, & Reddy, 2004; Sivaguru, Mander, Fried, & Punyasena, 2012). Alternative analyses, particularly regarding chemical composition of pollen such as measurement of proteins (Roulston, Cane, & Buchmann, 2000), carbohydrates (Speranza et al., 1997), and lipids (Piffanelli, Ross, & Murphy, 1998), are rarely conducted because they require complex sample preparation and laborious analysis. Recently, Fourier transform infrared spectroscopy (FTIR) has emerged as a significant breakthrough in pollen analysis as a precise fingerprint of the overall biochemical composition of pollen grain is provided (Bağcıoğlu, Zimmermann, & Kohler, 2015; Gottardini, Rossi, Cristofolini, & Benedetti, 2007; Jiang et al., 2015; Lahlali et al., 2014; Pappas, Tarantilis, Harizanis, & Polissiou, 2003; Zimmermann, 2010; Zimmermann, Bagcioglu, Sandt, & Kohler, 2015a; Zimmermann & Kohler, 2014; Zimmerman, Tafintseva, Bagcioglu, Hoegh Berdahl, & Kohler, 2016; Zimmermann, Tkalcec, Mesic, & Kohler, 2015b). Infrared spectra of pollen contain specific signals (i.e., vibrational frequencies of molecular bonds) that can be directly related to molecular functional groups and indirectly to biomolecules, such as lipids, proteins, carbohydrates, cell wall biopolymers, and other biochemical constituents. Moreover, FTIR can be combined with a microscope for focused measurement, thus providing single grain identification, as well as chemical imaging of pollen grain ultrastructure (Zimmermann et al., 2015a; Zimmerman et al., 2016). In general, FTIR enables fast and economical measurement of samples without any chemical pretreatment. The studies have shown that FTIR spectroscopy is a powerful method for pollen phenotyping because it provides precise identification and chemical characterization with respect to phylogeny and environmental stress (Bağcıoğlu, Kohler, Seifert, Kneipp, & Zimmermann, 2017; Jiang et al., 2015; Lahlali et al., 2014; Zimmermann & Kohler, 2014; Zimmermann et al., 2015b), including influence of anthropogenic stress on chemical composition of pollen (Depciuch, Kasprzyk, Roga, & Parlinska‐Wojtan, 2016; Depciuch, Kasprzyk, Sadik, & Parlinska‐Wojtan, 2017). However, the use of FTIR spectroscopy to study the genetic and phenotypically plastic components of the chemical composition of pollen and its potential in studies of evolution of male function has not been evaluated.

In this article, we develop an approach for studies of the adaptive role of the chemical composition of pollen based on FTIR spectroscopy. We cultivated three grass species to determine the degree and nature of variation in pollen chemical composition within and between species. We have investigated to what extent chemical composition of pollen is plastic in response to differing temperatures and nutrient levels. Moreover, we have provided spectral signatures, that are biochemically interpretable and related to phylogeny and environmental stress, by employing Sparse partial least‐squares regression (Karaman et al., 2013; Lê Cao, Rossouw, Robert‐Granié, & Besse, 2008; Zimmerman et al., 2016).

## 2 MATERIALS AND METHODS

### 2.1 Materials

#### 2.1.1 Study species

*Anthoxanthum odoratum* L. is a perennial species with caespitose growth (Clayton, Vorontsova, Harman, & Williamson, 2006). It is distributed throughout Europe and Asia, and it is introduced to Northern America (Clayton et al., 2006; Hulten & Fries, 1986; Tutin et al., 1980).

*Festuca ovina* L. is a perennial species with caespitose growth (Clayton et al., 2006). It is distributed in Northern and Central Europe, and it is introduced to Northern America, Far East, and New Zealand (Clayton et al., 2006; Hulten & Fries, 1986; Tutin et al., 1980).

*Poa alpina* L. is a perennial species with caespitose growth (Clayton et al., 2006). It grows in mountain pasture and exposed grassland. It is distributed in mountains in Europe, Asia, and North America and locally at low altitudes in the North (Clayton et al., 2006; Hulten & Fries, 1986; Tutin et al., 1980).

Seed of three populations from each of *P. alpina*,* A. odoratum*, and *F. ovina* was acquired from the Nordic Gene Bank. The populations were chosen to cover geographic and climatic variation. Seeds were sown in spring 2013 in Tjerbo Gartnerjord (Tjerbo, Rakkestad, Norway), and 15 individuals per species were grown outside at the Norwegian University of Life Sciences, Ås, Norway. After the summer, each individual was divided into four clones. During this period, plants were fertilized as needed with water containing 4% Yara Kristalon Indigo (Yara, Skøyen, Norway) and 3% YaraLiva calcium nitrate (Yara, Skøyen, Norway) adjusted to an electron conductivity of 1.5. To induce flowering, we vernalized the plants for 12 weeks at 4°C under short day length (8 hr). After vernalization, the four clones were subjected to four different treatments: high (20°C) and low temperature (14°C) in combination with high and low levels of nutrients (±NU). Plants from all treatments were grown under log day inductive conditions of 20 hr. The +NU plants were watered twice a week with nutrient containing water. The −NU plants received only water; thus, no extra fertilizer was added apart from what was in the soil at the start of the flowering period. Plants were randomized between and within treatments. Within each treatment, plants were rotated three times a week to avoid room‐effects. In total, 520 individuals were included in the flowering experiment, after removing misclassified individuals and some individuals with mold infection. A total of 460 individuals produced inflorescence, and, of those, 434 produced sufficient amount of pollen for spectroscopic analysis (at least 0.5 mg per individual). A total of 761 samples of pollen were collected in total (see Tables S1 and S2).

Georeferenced data for all species were downloaded from the Global Biodiversity Information Facility (http://www.gbif.org/, GBIF Secretariat, 2013). For each georeference, annual mean temperature (bio1), isothermality (bio3), temperature seasonality (bio4), max temperature of warmest month (bio5), temperature annual range (bio7), and mean temperature of warmest quarter (bio10) were extracted from Worldclim (http://www.worldclim.org/; Hijmans, Cameron, Parra, Jones, & Jarvis, 2005). Boxplots of each parameter and species were made using the boxplot function in R (R core team 2015) with outline set to FALSE.

### 2.2 Spectroscopic analysis

Three hundred and eighty pollen samples, each belonging to a different individual plant, were covered by the *main FTIR study* (see Table S3). The main study covered 140 samples of *A. odoratum* (44, 48, and 48 for populations France, Greece, and Finland, respectively), 96 samples of *F. ovina* (48, 32, and 16 for populations Sweden, Finland, and Italy, respectively), and 144 samples of *P. alpina* (48 samples for each of the populations: Sweden, Italy, and Norway). Samples were uniformly distributed, to cover each of the four growth conditions with a quarter of samples (95 samples in total per growth condition). In addition to the main FTIR study, an *FTIR timeline study* was conducted by covering nine additional pollen samples of the same individual plant (*F. ovina*, population Italy, 20°C with added nutrients). All 10 samples (one from the main study plus nine additional ones) were collected at different times (spanning 11 days in total).

For FTIR measurements, homogeneous suspensions of pollen samples were prepared. Approximately 1 mg of a pollen sample was transferred into 1.5‐ml microcentrifuge tube containing 500 μl of distilled H_2_O. The sample was sonicated in ice bath, by a 2 mm probe coupled to a Q55 Sonicator ultrasonic processor (QSonica, LLC, USA) under 100 % power. The sonication period was 2 min in total, with 30‐s intermission after the first minute of sonication to minimize the increase of temperature. Following the sonication, the sample was centrifuged with 13,000 rpm for 10 min, and the suspension was concentrated by removing 400 μl of supernatant. Of the remaining suspension, three aliquots, each containing 8 μl, were transferred onto IR‐light‐transparent silicon 384‐well microtiter plate (Bruker Optik GmbH, Germany). The microtiter plate was dried at room temperature for 1 hr at r.t. to create adequate films for FTIR measurement.

Infrared spectroscopy measurements were obtained using a HTS‐XT extension unit coupled to a TENSOR 27 spectrometer (both Bruker Optik GmbH, Germany). The systems are equipped with a globar mid‐IR source and a DTGS detector. The spectra were recorded in transmission mode, with a spectral resolution of 4 cm^−1^ and digital spacing of 0.964 cm^−1^. Background (reference) spectra of an empty well on a microtiter plate were recorded before each sample well measurement. The spectra were measured in the 4,000–500 cm^−1^ spectral range, with 32 scans for both background and sample spectra, and using an aperture of 5.0 mm.

Data acquisition and instrument control were carried out using the OPUS/LAB software (Bruker Optik GmbH, Germany).

### 2.3 Spectral preprocessing and data analysis

For the *main FTIR study*, the FTIR spectral data set consisted of 1,140 spectra, belonging to 380 pollen samples measured in three technical replicates. The entire spectral region of 4,000 to 500 cm^−1^ was used for data analysis. The spectra were preprocessed by taking the second derivative employing the Savitzky–Golay algorithm (Savitzky & Golay, 1964) with a polynomial of degree two and a windows size of 7 points, followed by extended multiplicative signal correction (EMSC) with linear and quadratic components (Zimmermann & Kohler, 2013). Finally, technical replicates were averaged resulting in 380 average spectra in total.

Classification and biochemical similarities between pollen samples were evaluated using principal component analysis (PCA), partial least‐squares regression (PLSR), and variability tests based on Pearson correlation coefficients and Fisher discriminant analysis. PLSR was used for building discrete and hierarchical classification schemes.

For the hierarchical classification, the complete data set was split into a training set, comprising 218 spectra, and a validation set, comprising 162 spectra. The calibration models were established using sparse partial least‐squares regression (SPLSR), an advancement of PLSR for variable selection that allows straightforward biochemical interpretation of spectral bands due the selection of a limited subset of variables in the modeling process (Lê Cao et al., 2008). The SPLSR models were established with 3‐block cross‐validation (CV) of randomly split samples for training the model. SPLSR applied to a whole spectrum removes variables in loading weight vectors, which are below a certain threshold. The threshold λ is defined by the desired number of variables to be neglected called degree of sparsity (Karaman et al., 2013). For each principal component, the degree of sparsity is optimized in a range between 90% and 99% of variables. The optimal degree of sparsity was defined by CV as the one which gave minimum misclassification rate (MCR). The additional details regarding SPLSR are presented in the Supporting information. The success rate (SR) was expressed in percentage and is defined as (1 − MCR) × 100. A hierarchical classification tree with three levels (species, population, growth conditions) was established with one model for each node, thus one model for the species, three models for the populations, and nine models for the clones, resulting in 13 models in total. Models were evaluated by an independent test set.

To evaluate the ability to detect an influence of environmental conditions on the plants and pollen, two classification models were established for each of the nine different populations: one classification model separating temperature and one classification model separating nutrition. As the average number of samples in each population was 42, an external validation could not be performed. Therefore, the statistical significance of the obtained models was evaluated by a permutation test (Lindgren, Hansen, Karcher, Sjostrom, & Eriksson, 1996; Szymanska, Saccenti, Smilde, & Westerhuis, 2012). The additional details regarding the permutation test are presented in the Supporting information.

To evaluate interaction effects of nutrient and temperature conditions, models were established for the nine different populations which classified into the four interaction groups.

Fisher discriminant analysis was performed on PCA scores of spectral data for each population separately. The between‐ and within‐class scatter matrices were calculated as follows: $S_{b}=\sum_{i}n_{i}\left(\mu -{\overset{¯}{x}}_{i}\right){\left(\mu -{\overset{¯}{x}}_{i}\right)}^{T}$ and $S_{w}=\sum_{i}\sum_{j}\left({\overset{¯}{x}}_{i}-x_{ij}\right){\left({\overset{¯}{x}}_{i}-x_{ij}\right)}^{T}$, where μ is a global mean, ${\overset{¯}{x}}_{i}$ is a group mean of group *i*,* n*_*i*_ is a number of samples in group *i*. The ratio, Fisher clustering coefficient (FCC), *S*_b_/*S*_w_ was used to evaluate the genotype‐based clustering of spectral data in the PC subspace.

For the *FTIR timeline study*, the FTIR spectral data set consisted of 75 spectra, belonging to 16 pollen samples of Italian *F. ovina*, population (from the main study) plus additional nine samples of the same plant collected at different time points. The data were preprocessed in the same way as for the main study, but without the averaging step. The variability between was estimated by calculating Pearson correlation coefficient (PCC) (Lee Rodgers & Nicewander, 1988) and was expressed as 1 − PCC × 10^−4^ for the spectral region 1,700–1,600 cm^−1^.

All preprocessing methods and data analyses were performed using in‐house developed routines written in MATLAB 2015a (The MathWorks, Natick, USA).

## 3 RESULTS

**Figure 1:**
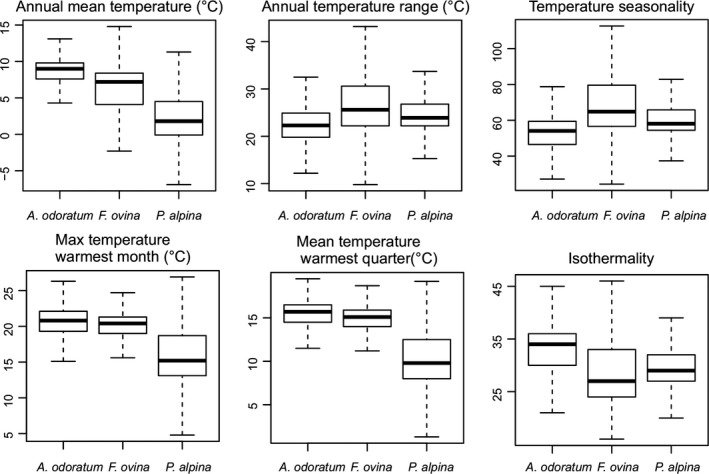
Variation in various climatic parameters between the distribution areas of *A. odoratum*,* F. ovina*, and *P. alpina*. The boxplots show median, lower and upper quartiles (25% and 75%), and minimum and maximum values. The boxplot is based on georeferences from GBIF (http://www.gbif.org/, GBIF Secretariat, 2013) and climatic data from Worldclim (Hijmans et al., 2005)

Comparing the climatic variables between distribution areas of the three species show that *P. alpina* has a lower annual mean temperature (bio1) than *F. ovina*, which in turn had lower annual mean temperature than *A. odoratum* (Figure 1). The growth season (bio5, bio10) of *P. alpina* was colder than those of *A. odoratum* and *F. ovina*. No trends could be seen for temperature annual range (bio7) and seasonality (bio3, bio7).

### 3.1 Growth and flowering of sampled plants

**Figure 2:**
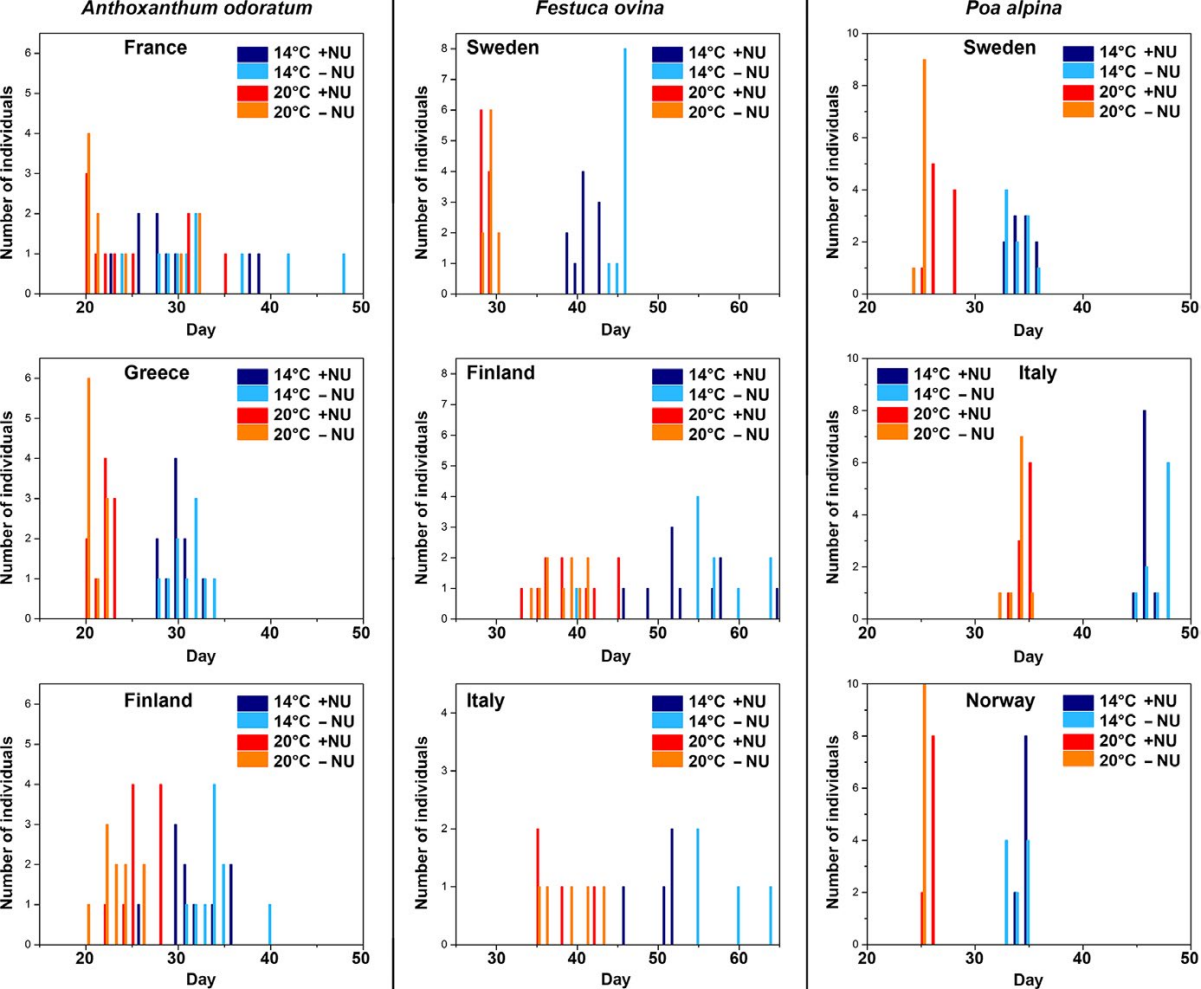
Pollen phenology for grass species and populations. The start of pollination is designated for first ten individuals per population; days are counted from the start of four different treatments (temperature and levels of nutrient)

The species showed different flowering phenologies: While *P. alpina* individuals were pollinating within 3–4 days, *A. odoratum* individuals were pollinating within several weeks and with almost continuous production of new inflorescence. As can be seen in Figure 2, higher temperature shifts start of pollination period by approx. 10 days. Nutrient regime does not have big impact, except for *F. ovina* in cold treatment, where low‐nutrient regime delays pollination season for approx. 5 days. *P. alpina* individuals have extremely synchronized pollination start. This is of importance because total pollination period for *P. alpina* is approx. 3–4 days. *A. odoratum* individuals have variable pollination start. This has not big impact because the individuals were pollinating throughout several weeks, and with almost continuous production of new inflorescence. *F. ovina* populations show both types of behavior.

### 3.2 FTIR measurement of pollen

The spectral variability of pollen samples was estimated by Pearson correlation coefficient (PCC) (Table 1). Variability was estimated by measurement of samples belonging to Italian population of *F. ovina*. Technical replicates refer to repeated FTIR measurements using the same pollen suspension, which was applied to different sample positions on the silicon well plate. The variability of the pollen composition for one individual during the pollination season was estimated based on spectral variability of samples belonging to the same plant (*F. ovina*, population Italy, 20°C with added nutrients), collected during 11 consecutive days, from the start to the end of pollination. As can be seen from the Table 1, the variability in pollen composition for this individual was slightly higher than variability within FTIR measurement (i.e., technical replicates) and considerably lower than variability for the whole population in the corresponding growth condition. The spectral variability for the whole population in all four growth conditions is two‐and‐a‐half times higher than variability within samples belonging to one individual plant.

**Table 1:** Variability within technical replicates and biological samples of *Festuca ovina* (Italy)

Type of variability	(1 − PCC) × 10^−4^
Technical replicates^a^	15 ± 7
Within *F. ovina* individual^a^	34
Within *F. ovina* (Italy), 20°C, N+	56
Within *F. ovina* (Italy)	85

a Based on measurements of samples from the *FTIR timeline study* (see Section 2).

### 3.3 Pollen composition and phylogeny

**Figure 3:**
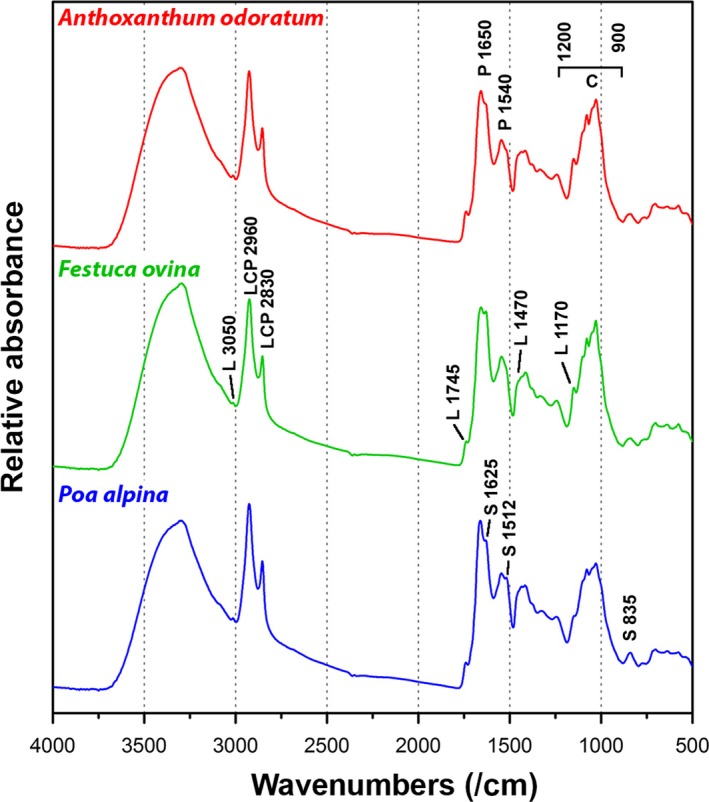
Representative spectra of pollen. All spectra belong to pollen samples collected from plants that were growing at 20°C in combination with high levels of nutrient. For better viewing, the spectra are offset; the marked bands are associated with molecular vibrations of (P) proteins, (L) lipids, (C) carbohydrates, and (S) sporopollenins

The representative FTIR spectra of pollen of the three species are shown in Figure 3. The spectra are dominated by signals associated with proteins, lipids, and carbohydrates. (Gottardini et al., 2007; Zimmermann et al., 2015b) The characteristic protein signals are at approx. 1,650 cm^−1^ (amide I:C = O stretch) and approx. 1,540 cm^−1^ (amide II: NH deformation and C–N stretch), carbohydrate at 1,200–900 cm^−1^ (C–O–C, C–C, and C–O stretching vibrations), and lipid signals at 1,745 (C=O stretch), 1,470 (CH_2_ deformation), and 1,170 cm^−1^ (C–C stretch). The major carbohydrate signals (1,200–1,000 cm^−1^) can be associated with cellulose and amylose (starch). In addition, the spectra show signals related to CH stretching at 3,050 cm^−1^ (=C–H stretch) and 2,960–2,830 cm^−1^ (C–H stretch in –CH_3_ and –CH_2_–) that will always be observed due to the presence of alkyl and alkenyl groups in cellular components like lipids, proteins, and carbohydrates. Finally, the spectra show signals associated with sporopollenins, a grain wall biopolymers based on phenylpropanoid acids (Bağcıoğlu et al., 2015; Zimmermann, 2010). The major sporopollenins signals (1,668, 1,658, 1,625, 1,512, 850, 835, and 815 cm^−1^) can be associated with ferulic and sinapic building blocks, although the presence of p‐coumaric building block cannot be excluded.

Hierarchical classification of spectral data was conducted on three levels by SPLSR. Success rates (SR) for identification were as follows: 1st level (species) SR = 99.4%, 2nd level (population) SR = 77.0%, and 3rd level (growth conditions) SR = 43.5%. Confusion matrices for all three levels are presented in Figs S1–S3.

The success rates for identification of samples obtained by SPLSR hierarchical classification of pollen FTIR spectral data can be seen in Table 2. The misclassification at species level occurred only for the Finish population of *F. ovina*. The plants from this population often produced quite small amount of pollen, thus resulting with suboptimal sample quantities for FTIR measurement. Consequently, the corresponding FTIR spectra had significantly lower absorbance values (approx. half of the average value for *A. odoratum* and *P. alpina* samples, Fig. S4). Therefore, this misclassification could be attributed to technical reasons. In general, all three species have distinguishing FTIR spectra that can be used for accurate identification.

**Table 2:** Success rates for identification of samples obtained by SPLSR hierarchical classification of pollen FTIR spectral data; results are based on validation set

Samples	Identification of species		Identification of populations (%)
*A. odoratum*, France	100%	100%	75
*A. odoratum*, Greece		100%	75
*A. odoratum*, Finland		100%	20
*F. ovina*, Sweden	98%	100%	85
*F. ovina*, Finland		94%	59
*F. ovina*, Italy		100%	100
*P. alpina*, Sweden	100%	100%	90
*P. alpina*, Italy		100%	100
*P. alpina*, Norway		100%	100

The regression coefficients for classification of species show that the classification is based on several species‐specific chemical fingerprints (Fig. S4). *P. alpina* has higher protein‐to‐carbohydrate ratio than the other two species. This feature is clearly seen in Figure 3 by comparing “protein” spectral regions (1,700–1,500 cm^−1^) and “carbohydrate” spectral regions (1,200–900 cm^−1^) of the three species. Differentiation between *A. odoratum* and the other two species is based on signals at 1,076 and 1,052 cm^−1^ that can be associated with higher concentration of amylose. In addition, *A. odoratum* has higher lipid‐to‐carbohydrate ratio than *F. ovina*. Differentiation between *P. alpina* and the other two species is based on the signal at 1,164 cm^−1^ that is probably associated with higher quantity of arabinoxylan. Moreover, ratios of sporopollenins‐related signals at 1,512, 1,625, 1,658, and 1,668 cm^−1^ for *A. odoratum* and *F. ovina* indicate differences in sporopollenin composition. Finally, all three species show differences in the amount and chemical composition of lipids, as indicated by signals at 2,923, 1,745, and 1,712 cm^−1^.

Chemical differences between populations are species specific (Fig. S5). While differences between populations of *P. alpina* are statistically significant, enabling accurate identification of all three populations, the differences between either *A. odoratum* or *F. ovina* populations are less prominent (Table 2). Regarding *P. alpina*, the Norwegian population has higher ratio of carbohydrates to lipids, compared to other two populations. These carbohydrate signals, in 1,200–900 cm^−1^ region, can be associated with amylose. In addition, differentiation of *P. alpina* populations is based on extensive differences in amide I and amide II protein bands.

### 3.4 Pollen composition and growth conditions

Table 3 shows the *p*‐values for environmental effect on pollen FTIR spectral data (Figs S6–S8). Eight populations, including all populations of *P. alpina*, show statistically significant (*p* < .05) correlation between pollen composition and temperature conditions during plants growth. In addition, six populations show statistically significant correlation between pollen composition and nutrient conditions during plants growth, while in the remaining three cases, the correlation is high (*p* < .1), but not statistically significant. Italian population of *F. ovina* is the only population that has not shown high correlation between pollen composition and growth conditions. As stated previously, this could be due to lower spectral quality and smaller number of samples compared to an average population. To evaluate the influence of all four growth conditions on pollen composition, nine other models were established. The models were of successful quality, but on average worse than the separate temperature and nutrition classification models.

**Table 3:** *p*‐values and success rates (SR) for environmental effect on pollen FTIR spectral data; results are based on SPLSR calibration set

Samples	Temperature		Nutrients		Growth conditions^a^	
	*p*‐value	SR (%)	*p*‐value	SR (%)	*p*‐value	SR (%)
*A. odoratum*, France	**.001**	90.9	**.002**	75.0	**.001**	79.5
*A. odoratum*, Greece	**.001**	79.2	**.004**	75.0	**.001**	66.7
*A. odoratum*, Finland	**.001**	77.1	.055	62.5	**.001**	66.7
*F. ovina*, Sweden	**.001**	77.1	**.002**	72.9	**.001**	58.3
*F. ovina*, Finland	**.009**	75.0	.074	65.6	**.005**	43.8
*F. ovina*, Italy	.23	62.5	.068	75.0	.021	43.8
*P. alpina*, Sweden	**.001**	95.7	**.013**	70.2	**.001**	76.6
*P. alpina*, Italy	**.001**	97.9	**.001**	91.7	**.001**	85.4
*P. alpina*, Norway	**.001**	89.6	**.001**	85.4	**.001**	79.2

a For SPLSR models for separation of all four environmental conditions with 10 principal components (detailed results are in the Supporting information). Bold: *p*‐values ≤ 0.05.

The chemical interpretation of spectral differences regarding growth conditions should be done with caution, because the differences are small, population specific, and highly complex (Figs S9–S11). Nevertheless, some general conclusions can be made. Regarding nutrient conditions, the main spectral differences are associated with changes in sporopollenin (1,626 and 1,510) and lipid signals (1,740–1,710, 1,473, and 1,150 cm^−1^). Moreover, more nutrients result with higher protein‐to‐carbohydrate ratio, as indicated by changes in protein signals (at approx. 1,660 and 1,530 cm^−1^) and starch signals (at approx. 1,070 and 1,050 cm^−1^). These differences are consistent for both *A. odoratum* and *P. alpina*. Regarding temperature conditions, the differences are species specific. Carbohydrate signals at 1,083 and 1,023 cm^−1^, probably related to amylose, are higher for *A. odoratum* samples grown in cold conditions than for warmer conditions. In case of *P. alpina*, the protein‐related amide signals are higher for samples grown in warm conditions than for colder conditions.

### 3.5 Phenotypic plasticity and rigidity of pollen composition

Table 3 shows that pollen compositions of the majority of populations are quite plastic, particularly regarding temperature conditions. However, it is questionable if the remaining cases, for which there is no significant correlation with growth conditions, are showing opposite behavior, that is, phenotypic rigidity. To test this, we have performed clustering analysis based on genotypes (i.e., genet with four ramets growing under different conditions), to estimate phenotypic rigidity based on Fisher clustering coefficient (FCC). The high FCC values indicate clustering based on genotype (i.e., high phenotypic rigidity), while low values indicate either high plasticity or random variability.

The Fisher clustering coefficient (FCC) for estimating clustering based on genotype can be seen in Table 4. Finish population of *A. odoratum* has the highest FCC value, strongly implying high phenotypic rigidity (Fig. S12). The FCC values for Finish population of *F. ovina* are relatively high, thus potentially indicating high phenotypic rigidity for this population as well. However, this could be due to, previously mentioned, lower spectral quality of data for this population, so random variability cannot be excluded. The lowest FCC values were obtained for *P. alpina* populations, thus validating notion that this species has high plasticity of pollen chemical composition (Fig. S12).

**Table 4:** Fisher clustering coefficient (FCC) for estimating clustering based on genotype

Samples	FCC
*A. odoratum*, France	0.26
*A. odoratum*, Greece	1.09
*A. odoratum*, Finland	10.29
*F. ovina*, Sweden	0.60
*F. ovina*, Finland	1.29
*F. ovina*, Italy	0.16
*P. alpina*, Sweden	0.08
*P. alpina*, Italy	0.04
*P. alpina*, Norway	0.01

## 4 DISCUSSION

### 4.1 FTIR reveal inter‐ and intraspecific differences

The three closely related grass species have highly specific FTIR spectroscopy profiles (Table 2). Their spectra were dominated by proteins, lipids, carbohydrates, and sporopollenin (Figure 3). Moreover, we detected within‐species variation in pollen composition (Table 2), and interestingly, the amount of within‐species variation in pollen composition was species specific. In particular, *P. alpina* had highly specific profiles for the three populations whereas classification of populations in *A. odoratum* and *F. ovina* was less precise. Our previous study has shown that high‐throughput FTIR measurement variations due to sample preparation, microplate holders, and instrumentation are low (Bağcıoğlu et al., 2017). In this study, we have tested variation for one individual during the intraseasonal pollination. The results indicate that pollen composition of the individual grass plant is quite invariant (Table 1), and thus, the approach enables precise measurement of individual genotypes. Furthermore, sparse PLSR allows straightforward biochemical interpretation of spectral bands due the selection of a limited subset of variables in the modeling process (see details in the Supporting information).

### 4.2 Growth conditions influence the chemical composition of pollen

The spectroscopic analysis of *P. alpina* shows that, pollen matured at high temperature had a higher level of proteins, whereas colder temperature gave higher levels of lipids and carbohydrates (Table 3 and the Supporting information). Similar results were found for pollen of *Petunia hybrida* sired at lower temperatures (19.5°C vs 25.5°C), which showed a reduced level of protein and low molecular weight carbohydrates (Van Herpen, 1986; Van Herpen & Linskens, 1981), whereas amount of lipids were the same.

More nutrients, including nitrogen and phosphorus, gave higher protein‐to‐carbohydrate ratio in both *A. odoratum* and *P. alpina*. A relationship between nutrition (phosphorous) and fitness was established in a study of *Cucurbita pepo* where plants in nutrient‐rich soils showed higher pollen production per flower and larger pollen grain size (Lau & Stephenson, 1994). This gave a fitness advantage as pollen from high phosphorous treatment sired significantly more mature seeds than pollen from nutrient poor soils. It is possible that a similar fitness advantage can be found in *A. odoratum* and *P. alpina* grown under high nutrient conditions in our study, but this needs further examination. Furthermore, we also demonstrate that changes in lipids and sporopollenin are associated with different nutrient regimes of parent plants. Lipids play an important role in pollen germination (Dickinson, Elleman, & Doughty, 2000) and are essential for the pollen tube to penetrate the stigma. However, the nature of changes in lipids and sporopollenin in our experiment are more diffuse and we might only speculate that it is adaptive.

Finally, decrease in amylase‐related signals with temperature in *A. odoratum* could indicate acclimation to warmer conditions (Figure 1). The decreased concentration of amylose could indicate that pollen starch is partly hydrolyzed into low molecular weight carbohydrates, such as sucrose, glucose, and fructose. These soluble carbohydrates are related to pollen longevity through regulation of water content (Hesse, 2006). The pollen of grasses is not dehydrated upon release like most plant species and looses water quickly (Bassani, Pacini, & Franchi, 1994).

### 4.3 Species‐specific responses indicate adaptation to different niches

*Festuca ovina* and *A. odoratum* are widespread Eurasian species growing in a wide range of environments, from lowland to alpine regions. *P. alpina* has a restricted distribution and an alpine and subarctic distribution. Corresponding to this, *A. odoratum* and *F. ovina* are more of generalists (Dennis, Dapporto, Fattorini, & Cook, 2011) than *P. alpina*, although all species are plastic in response to different environments (Table 3; Figure 2). The differences in niches are best reflected in the temperature variables (Figure 1). Maximum temperature in the warmest month and the mean temperature of the warmest quarter describe the growing season, which is cool and short (mean temperature for the warmest quarter is 10°C, which is generally recognized as the lower limit that allows growth to persist) for *P. alpina*. Accordingly, the pollination season of *P. alpina* was short and concluded in 3–4 days (Figure 2). *F. ovina* and *A. odoratum* mostly had prolonged pollination continuing over several weeks, and *A. odoratum* continuously produced new inflorescences. *P. alpina* has a higher protein‐to‐carbohydrate ratio than *A. odoratum* and *F. ovina* reflecting the advantage of nutrient‐rich pollen of high quality in a short growing season. *P. alpina* also had the highest level of plasticity of the species which may be a specialization to maximize reproductive output in the short growing season often with unpredictable and unstable climatic conditions. However, to conclude whether or not plasticity is adaptive and enhance fitness, further research is needed.

As opposed to *P. alpina*,* A. odoratum* and *F. ovina* produce more pollen over longer periods of time (Figure 2), invest less nitrogen nutrients in each pollen grain (i.e., higher carbohydrate‐to‐protein ratio), and pollen composition is more invariant between populations and environments. Taken together, this reflects more of a generalist strategy with a trade‐off between quality and quantity giving more, rather than, nutrient‐rich, high‐quality pollen. In contrast, *P. alpina* invests more in pollen quality in terms of higher protein‐to‐carbohydrate ratio and plastic responses, but produces less pollen grains in a short time span. Within species, all populations of *P. alpina* had specific spectra whereas the differences were less prominent in *A. odoratum* and *F. ovina*. The distinct profiles of the *P. alpina* populations may be related to the discontinuous distribution pattern of the species (Fjellheim, Tanhuanpaa, Marum, Manninen, & Rognli, 2015), possibly also in combination with limited pollen production period (Figure 2). This will prevent gene flow between populations which over time will diverge. The opposite is the case for *F. ovina* and *A. odoratum* which have enormous potential for gene flow between populations through prolonged pollen production period and continuous geographic distribution (Hulten & Fries, 1986). Norwegian populations of *P. alpina* are genetically more divergent than populations of *F. ovina*, most likely reflecting a difference in gene flow (Jørgensen et al., 2016). The outcrossing temperate forage grass *Phleum pratensis* is a species very similar in biology and distribution as *F. ovina* and *A. odoratum* (Hulten & Fries, 1986) and shows no genetic structure in the entire distribution area reflecting large amount of gene flow (Fjellheim et al., 2015). It is likely that *F. ovina* and *A. odoratum* also exhibit large amount of gene flow which will counteract divergence between populations.

## 5 CONCLUDING REMARKS

In this study, we show that high‐throughput FTIR spectroscopy in combination with sparse PLSR offers fast, economical, and accurate classification of genetic and plastic responses in pollen chemical composition to environmental conditions. Our results indicate that pollen composition has an adaptive role and should be investigated in more detail in future studies. Prospected climate change may influence the chemical composition and quality of pollen; however, the potential for species to respond to those changes through adaptive evolution remains unexplored. More studies are needed to study if plasticity in pollen composition is adaptive and to study if pollen composition influences fitness under different conditions. FTIR coupled with controlled environment studies of plants and direct effects on fitness of pollen with different composition has the potential to shed new light on the evolutionary significance of plant male function and adaptation.

## CONFLICT OF INTEREST

None declared.

## AUTHOR CONTRIBUTIONS

AK, BZ, MO, and SF conceived the research idea. BZ and SF designed the experiments. BZ and MB performed the experiments. AK, BZ, MB, and VT analyzed the data. BZ, MO, and SF wrote the article. AK, MB, and VT discussed and revised the article.

## DATA ACCESSIBILITY

Data are available from the Dryad Digital Repository: https://doi.org/10.5061/dryad.2mm71.
