# Supplementary material for: A high‐throughput FTIR spectroscopy approach to assess adaptive variation in the chemical composition of pollen
Source: Ecol Evol. 2017 Nov 9;7(24):10839–49. doi: 10.1002/ece3.3619 (PMC5743575; doi:10.1002/ece3.3619)
Supplement: Supplementary file 1 [file ECE3-7-10839-s001.pdf]

Supporting Information

A high-throughput FTIR spectroscopy approach to assess adaptive variation in the chemical composition of pollen

Boris Zimmermann[1*], Murat Bağcıoğlu[1], Valeria Tafinstseva[1], Achim Kohler[1,2], Mikael Ohlson[3], Siri Fjellheim[4*]

[1]Faculty of Life Science and Technology, Norwegian University of Life Sciences, Ås, Norway
[2]Nofima AS, Ås, Norway
[3]Faculty of Environmental Sciences and Natural Resource Management, Norwegian University of Life Sciences, Ås, Norway
[4]Faculty of Biosciences, Norwegian University of Life Sciences, Ås, Norway

*Corresponding authors:
Siri Fjellheim
Tel: +47 6723 2801
Fax: +47 6496 5001
E-mail: siri.fjellheim@nmbu.no

Boris Zimmermann
Tel.: +47 6723 1576
Fax: +47 6496 5001
E-mail: boris.zimmermann@nmbu.no

**Table of Contents**	**Page**
Sample set	S2
Sparse partial least squares regression and permutation test	S4
Hierarchical classification: confusion matrices	S5
Hierarchical classification: regression coefficients	S8
Classification based on growth conditions: confusion matrices	S12
Classification based on growth conditions: regression coefficients	S21
Principal component analysis showing clustering based on genotype	S30

	Anthoxanthum odoratum			*Festuca ovina*			*Poa alpina*		
	France	Greece	Finland	Sweden	Finland	Italy	Sweden	Italy	Norway
14 °C +NU	12	15	15	15	15	15	15	14	14
14 °C - NU	12	15	15	15	15	15	15	14	14
20 °C +NU	12	15	15	15	15	15	15	14	14
20 °C - NU	12	15	15	15	15	15	15	14	14
total	48	60	60	60	60	60	60	56	56

Table S1. Number of individual plants per growth condition per population. Not all plants created inflorescence, and not all inflorescence created enough amount of pollen (see Table S2)

	Anthoxanthum odoratum			*Festuca ovina*			*Poa alpina*		
	France	Greece	Finland	Sweden	Finland	Italy	Sweden	Italy	Norway
14 °C +NU	11 (20)	14 (26)	15 (26)	15 (39)	8 (14)	4 (8)	15 (36)	12 (20)	14 (34)
14 °C - NU	11 (19)	14 (28)	15 (28)	15 (20)	8	4 (6)	15 (28)	14 (18)	14 (39)
20 °C +NU	11 (19)	14 (21)	13 (16)	15 (21)	8 (20)	4 (17)	15 (19)	14 (32)	14 (23)
20 °C - NU	11 (21)	14 (28)	14 (20)	15 (19)	8	4 (7)	15 (25)	13 (15)	14 (25)
4 clones	11	14	13	15	5	3	15	11	14

Table S2. Number of sampled individuals, and total number of pollen samples (in parentheses). Last row designates number of genotypes for which all four clones were sampled.

	Anthoxanthum odoratum			*Festuca ovina*			*Poa alpina*		
	France	Greece	Finland	Sweden	Finland	Italy	Sweden	Italy	Norway
14 °C +NU	11	12	12	12	8	4	12	12	12
14 °C - NU	11	12	12	12	8	4	12	12	12
20 °C +NU	11	12	12	12	8	4*	12	12	12
20 °C - NU	11	12	12	12	8	4	12	12	12
Total	44	48	48	48	32	16	48	48	48

Table S3. List of pollen samples covered by *main FTIR study*. *9 additional pollen samples of the same individual plant, collected at different times, were covered by *FTIR timeline study*.

Sparse partial least squares regression and permutation test

Sparse partial least squares regression

For each principal component the degree of sparsity is optimized in a range between 90 and 99 percent of variables. The optimal degree of sparsity was defined by CV as the one which gave minimum misclassification rate (MCR). The optimal number of SPLSR components was defined by CV in the following way: We calculate models with 1 to 25 SPLSR components, and the optimal number of components corresponds to the model which did not give significantly higher MCR than the minimum MCR (significance was evaluated by a binomial test; function binocdf in Matlab). The success rate (SR) was expressed in percentage and is defined as (1-MCR) ×100.

Permutation test

In each step of a permutation test, the labels of the samples are permuted randomly and a new classification model is established. A distribution of the null hypothesis, H_0, is obtained by performing N=1000 permutations. A P-value is calculated by comparing permuted MCR, MCR_p, to the original non-permuted MCR: $p = (1 + \#(MCR_p \leq MCR))/N$. For estimating the MCR, cross-validation, with 5-block and 6-block Venetian blinds was performed for temperature and nutrient classification respectively.

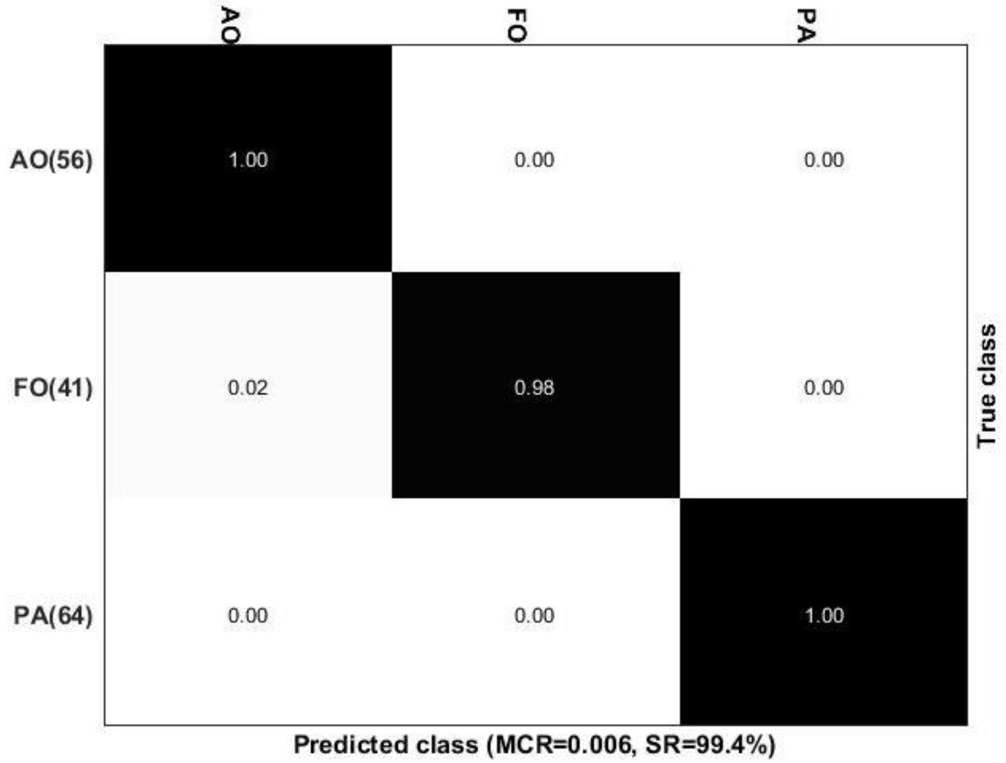

Fig. S1 Confusion matrix for hierarchical classification for 1st level (species); AO: *Anthoxanthum odoratum*, FO: *Festuca ovina*, PA: *Poa alpina*; number of spectra in parenthesis; MCR: misclassification rate, SR: success rate.

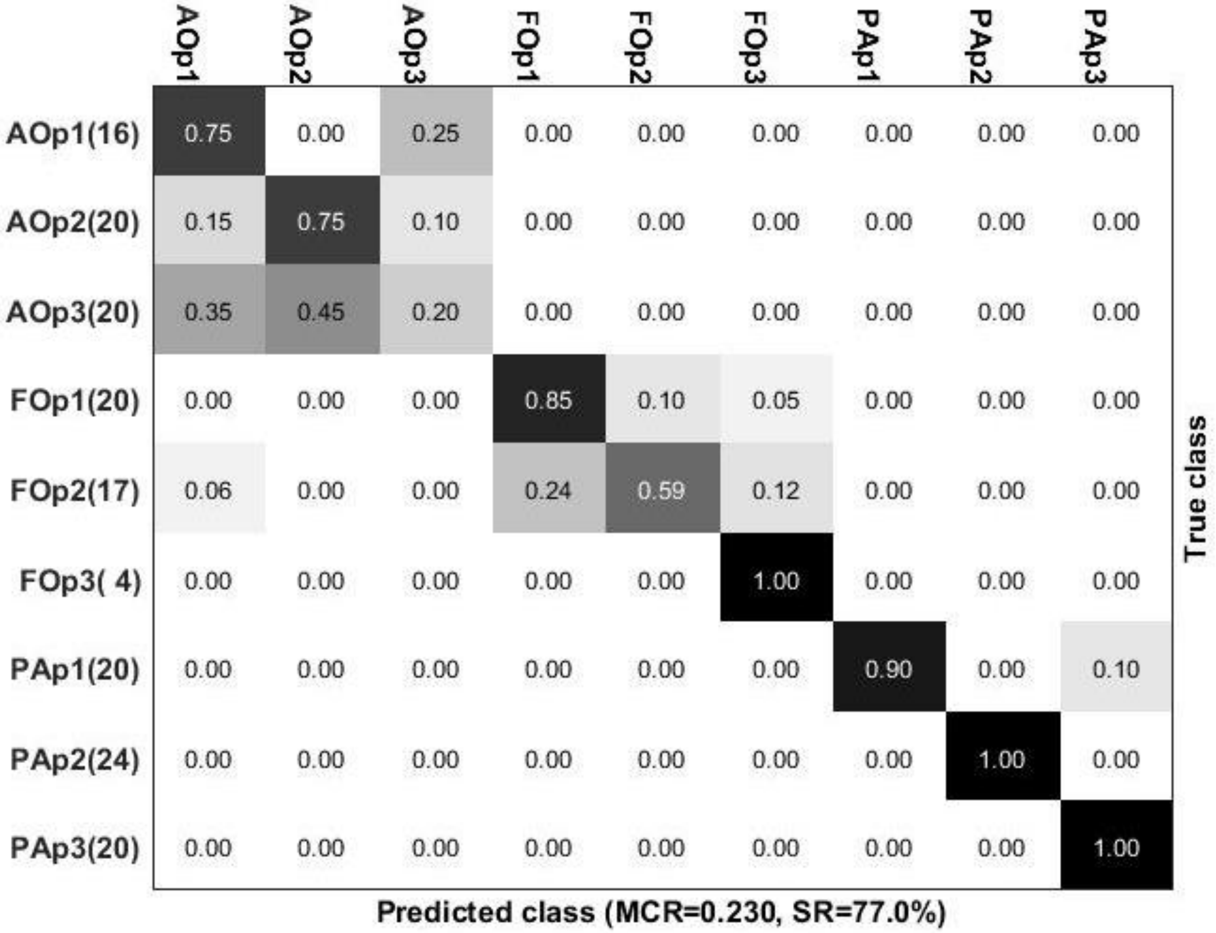

Fig. S2 Confusion matrix for hierarchical classification for 2nd level (population); AO: *Anthoxanthum odoratum* (p1: France, p2: Greece, p3: Finland), FO: *Festuca ovina* (p1: Sweden, p2: Finland, p3: Italy) PA: *Poa alpina* (p1: Sweden; p2: Italy, p3: Norway); number of spectra in parenthesis; MCR: misclassification rate, SR: success rate.

True class	AOp1c1	AOp1c2	AOp1c3	AOp1c4	AOp2c1	AOp2c2	AOp2c3	AOp2c4	AOp3c1	AOp3c2	AOp3c3	AOp3c4	FOp1c1	FOp1c2	FOp1c3	FOp1c4	FOp2c1	FOp2c2	FOp2c3	FOp2c4	FOp3c1	FOp3c2	FOp3c3	FOp3c4	PAp1c1	PAp1c2	PAp1c3	PAp1c4	PAp2c1	PAp2c2	PAp2c3	PAp2c4	PAp3c1	PAp3c2	PAp3c3	PAp3c4
AOp1c1(4)	0.50	0.25	0.00	0.00	0.00	0.00	0.00	0.00	0.00	0.25	0.00	0.00	0.00	0.00	0.00	0.00	0.00	0.00	0.00	0.00	0.00	0.00	0.00	0.00	0.00	0.00	0.00	0.00	0.00	0.00	0.00	0.00	0.00	0.00	0.00	0.00
AOp1c2(4)	0.50	0.50	0.00	0.00	0.00	0.00	0.00	0.00	0.00	0.00	0.00	0.00	0.00	0.00	0.00	0.00	0.00	0.00	0.00	0.00	0.00	0.00	0.00	0.00	0.00	0.00	0.00	0.00	0.00	0.00	0.00	0.00	0.00	0.00	0.00	0.00
AOp1c3(4)	0.50	0.00	0.25	0.00	0.00	0.00	0.00	0.00	0.00	0.25	0.00	0.00	0.00	0.00	0.00	0.00	0.00	0.00	0.00	0.00	0.00	0.00	0.00	0.00	0.00	0.00	0.00	0.00	0.00	0.00	0.00	0.00	0.00	0.00	0.00	0.00
AOp1c4(4)	0.50	0.00	0.00	0.00	0.00	0.00	0.00	0.00	0.00	0.00	0.00	0.50	0.00	0.00	0.00	0.00	0.00	0.00	0.00	0.00	0.00	0.00	0.00	0.00	0.00	0.00	0.00	0.00	0.00	0.00	0.00	0.00	0.00	0.00	0.00	0.00
AOp2c1(5)	0.00	0.00	0.00	0.00	0.40	0.20	0.00	0.20	0.20	0.00	0.00	0.00	0.00	0.00	0.00	0.00	0.00	0.00	0.00	0.00	0.00	0.00	0.00	0.00	0.00	0.00	0.00	0.00	0.00	0.00	0.00	0.00	0.00	0.00	0.00	0.00
AOp2c2(5)	0.00	0.20	0.00	0.00	0.00	0.20	0.20	0.40	0.00	0.00	0.00	0.00	0.00	0.00	0.00	0.00	0.00	0.00	0.00	0.00	0.00	0.00	0.00	0.00	0.00	0.00	0.00	0.00	0.00	0.00	0.00	0.00	0.00	0.00	0.00	0.00
AOp2c3(5)	0.00	0.00	0.20	0.00	0.00	0.00	0.60	0.20	0.00	0.00	0.00	0.00	0.00	0.00	0.00	0.00	0.00	0.00	0.00	0.00	0.00	0.00	0.00	0.00	0.00	0.00	0.00	0.00	0.00	0.00	0.00	0.00	0.00	0.00	0.00	0.00
AOp2c4(5)	0.20	0.00	0.00	0.00	0.00	0.00	0.20	0.40	0.00	0.00	0.00	0.20	0.00	0.00	0.00	0.00	0.00	0.00	0.00	0.00	0.00	0.00	0.00	0.00	0.00	0.00	0.00	0.00	0.00	0.00	0.00	0.00	0.00	0.00	0.00	0.00
AOp3c1(5)	0.00	0.00	0.00	0.00	0.00	0.00	0.80	0.00	0.00	0.20	0.00	0.00	0.00	0.00	0.00	0.00	0.00	0.00	0.00	0.00	0.00	0.00	0.00	0.00	0.00	0.00	0.00	0.00	0.00	0.00	0.00	0.00	0.00	0.00	0.00	0.00
AOp3c2(5)	0.20	0.00	0.00	0.20	0.00	0.00	0.40	0.00	0.00	0.20	0.00	0.00	0.00	0.00	0.00	0.00	0.00	0.00	0.00	0.00	0.00	0.00	0.00	0.00	0.00	0.00	0.00	0.00	0.00	0.00	0.00	0.00	0.00	0.00	0.00	0.00
AOp3c3(5)	0.20	0.00	0.20	0.20	0.00	0.00	0.00	0.00	0.00	0.20	0.00	0.20	0.00	0.00	0.00	0.00	0.00	0.00	0.00	0.00	0.00	0.00	0.00	0.00	0.00	0.00	0.00	0.00	0.00	0.00	0.00	0.00	0.00	0.00	0.00	0.00
AOp3c4(5)	0.20	0.00	0.20	0.00	0.00	0.00	0.40	0.20	0.00	0.00	0.00	0.00	0.00	0.00	0.00	0.00	0.00	0.00	0.00	0.00	0.00	0.00	0.00	0.00	0.00	0.00	0.00	0.00	0.00	0.00	0.00	0.00	0.00	0.00	0.00	0.00
FOp1c1(5)	0.00	0.00	0.00	0.00	0.00	0.00	0.00	0.00	0.00	0.00	0.00	0.00	0.20	0.00	0.20	0.60	0.00	0.00	0.00	0.00	0.00	0.00	0.00	0.00	0.00	0.00	0.00	0.00	0.00	0.00	0.00	0.00	0.00	0.00	0.00	0.00
FOp1c2(5)	0.00	0.00	0.00	0.00	0.00	0.00	0.00	0.00	0.00	0.00	0.00	0.00	0.40	0.00	0.60	0.00	0.00	0.00	0.00	0.00	0.00	0.00	0.00	0.00	0.00	0.00	0.00	0.00	0.00	0.00	0.00	0.00	0.00	0.00	0.00	0.00
FOp1c3(5)	0.00	0.00	0.00	0.00	0.00	0.00	0.00	0.00	0.00	0.00	0.00	0.00	0.00	0.20	0.40	0.20	0.20	0.00	0.00	0.00	0.00	0.00	0.00	0.00	0.00	0.00	0.00	0.00	0.00	0.00	0.00	0.00	0.00	0.00	0.00	0.00
FOp1c4(5)	0.00	0.00	0.00	0.00	0.00	0.00	0.00	0.00	0.00	0.00	0.00	0.00	0.00	0.00	0.00	0.60	0.00	0.20	0.00	0.00	0.00	0.00	0.00	0.20	0.00	0.00	0.00	0.00	0.00	0.00	0.00	0.00	0.00	0.00	0.00	0.00
FOp2c1(5)	0.00	0.20	0.00	0.00	0.00	0.00	0.00	0.00	0.00	0.00	0.00	0.00	0.00	0.00	0.20	0.20	0.00	0.40	0.00	0.00	0.00	0.00	0.00	0.00	0.00	0.00	0.00	0.00	0.00	0.00	0.00	0.00	0.00	0.00	0.00	0.00
FOp2c2(3)	0.00	0.00	0.00	0.00	0.00	0.00	0.00	0.00	0.00	0.00	0.00	0.00	0.00	0.00	0.00	0.00	0.00	1.00	0.00	0.00	0.00	0.00	0.00	0.00	0.00	0.00	0.00	0.00	0.00	0.00	0.00	0.00	0.00	0.00	0.00	0.00
FOp2c3(4)	0.00	0.00	0.00	0.00	0.00	0.00	0.00	0.00	0.00	0.00	0.00	0.00	0.00	0.00	0.00	0.50	0.00	0.25	0.25	0.00	0.00	0.00	0.00	0.00	0.00	0.00	0.00	0.00	0.00	0.00	0.00	0.00	0.00	0.00	0.00	0.00
FOp2c4(5)	0.00	0.00	0.00	0.00	0.00	0.00	0.00	0.00	0.00	0.00	0.00	0.00	0.00	0.00	0.00	0.00	0.00	0.00	0.00	0.60	0.00	0.00	0.00	0.40	0.00	0.00	0.00	0.00	0.00	0.00	0.00	0.00	0.00	0.00	0.00	0.00
FOp3c1(1)	0.00	0.00	0.00	0.00	0.00	0.00	0.00	0.00	0.00	0.00	0.00	0.00	0.00	0.00	0.00	0.00	0.00	0.00	0.00	0.00	0.00	0.00	0.00	1.00	0.00	0.00	0.00	0.00	0.00	0.00	0.00	0.00	0.00	0.00	0.00	0.00
FOp3c2(1)	0.00	0.00	0.00	0.00	0.00	0.00	0.00	0.00	0.00	0.00	0.00	0.00	0.00	0.00	0.00	0.00	0.00	0.00	0.00	0.00	0.00	1.00	0.00	0.00	0.00	0.00	0.00	0.00	0.00	0.00	0.00	0.00	0.00	0.00	0.00	0.00
FOp3c3(1)	0.00	0.00	0.00	0.00	0.00	0.00	0.00	0.00	0.00	0.00	0.00	0.00	0.00	0.00	0.00	0.00	0.00	0.00	0.00	0.00	0.00	0.00	0.00	1.00	0.00	0.00	0.00	0.00	0.00	0.00	0.00	0.00	0.00	0.00	0.00	0.00
FOp3c4(1)	0.00	0.00	0.00	0.00	0.00	0.00	0.00	0.00	0.00	0.00	0.00	0.00	0.00	0.00	0.00	0.00	0.00	0.00	0.00	0.00	0.00	1.00	0.00	0.00	0.00	0.00	0.00	0.00	0.00	0.00	0.00	0.00	0.00	0.00	0.00	0.00
PAp1c1(5)	0.00	0.00	0.00	0.00	0.00	0.00	0.00	0.00	0.00	0.00	0.00	0.00	0.00	0.00	0.00	0.00	0.00	0.00	0.00	0.00	0.00	0.00	0.00	0.00	0.40	0.20	0.20	0.00	0.00	0.00	0.00	0.00	0.00	0.00	0.20	0.00
PAp1c2(5)	0.00	0.00	0.00	0.00	0.00	0.00	0.00	0.00	0.00	0.00	0.00	0.00	0.00	0.00	0.00	0.00	0.00	0.00	0.00	0.00	0.00	0.00	0.00	0.00	0.20	0.80	0.00	0.00	0.00	0.00	0.00	0.00	0.00	0.00	0.00	0.00
PAp1c3(5)	0.00	0.00	0.00	0.00	0.00	0.00	0.00	0.00	0.00	0.00	0.00	0.00	0.00	0.00	0.00	0.00	0.00	0.00	0.00	0.00	0.00	0.00	0.00	0.00	0.00	0.00	0.80	0.20	0.00	0.00	0.00	0.00	0.00	0.00	0.00	0.00
PAp1c4(5)	0.00	0.00	0.00	0.00	0.00	0.00	0.00	0.00	0.00	0.00	0.00	0.00	0.00	0.00	0.00	0.00	0.00	0.00	0.00	0.00	0.00	0.00	0.00	0.00	0.00	0.20	0.20	0.40	0.00	0.00	0.00	0.00	0.00	0.00	0.20	0.00
PAp2c1(6)	0.00	0.00	0.00	0.00	0.00	0.00	0.00	0.00	0.00	0.00	0.00	0.00	0.00	0.00	0.00	0.00	0.00	0.00	0.00	0.00	0.00	0.00	0.00	0.00	0.00	0.00	0.00	0.00	0.67	0.17	0.17	0.00	0.00	0.00	0.00	0.00
PAp2c2(6)	0.00	0.00	0.00	0.00	0.00	0.00	0.00	0.00	0.00	0.00	0.00	0.00	0.00	0.00	0.00	0.00	0.00	0.00	0.00	0.00	0.00	0.00	0.00	0.00	0.00	0.00	0.00	0.00	0.17	0.67	0.00	0.17	0.00	0.00	0.00	0.00
PAp2c3(6)	0.00	0.00	0.00	0.00	0.00	0.00	0.00	0.00	0.00	0.00	0.00	0.00	0.00	0.00	0.00	0.00	0.00	0.00	0.00	0.00	0.00	0.00	0.00	0.00	0.00	0.00	0.00	0.00	0.00	0.00	0.83	0.17	0.00	0.00	0.00	0.00
PAp2c4(6)	0.00	0.00	0.00	0.00	0.00	0.00	0.00	0.00	0.00	0.00	0.00	0.00	0.00	0.00	0.00	0.00	0.00	0.00	0.00	0.00	0.00	0.00	0.00	0.00	0.00	0.00	0.00	0.00	0.00	0.00	0.50	0.50	0.00	0.00	0.00	0.00
PAp3c1(5)	0.00	0.00	0.00	0.00	0.00	0.00	0.00	0.00	0.00	0.00	0.00	0.00	0.00	0.00	0.00	0.00	0.00	0.00	0.00	0.00	0.00	0.00	0.00	0.00	0.00	0.00	0.00	0.00	0.00	0.00	0.00	0.00	0.80	0.00	0.00	0.20
PAp3c2(5)	0.00	0.00	0.00	0.00	0.00	0.00	0.00	0.00	0.00	0.00	0.00	0.00	0.00	0.00	0.00	0.00	0.00	0.00	0.00	0.00	0.00	0.00	0.00	0.00	0.00	0.00	0.00	0.00	0.00	0.00	0.00	0.00	0.20	0.80	0.00	0.00
PAp3c3(5)	0.00	0.00	0.00	0.00	0.00	0.00	0.00	0.00	0.00	0.00	0.00	0.00	0.00	0.00	0.00	0.00	0.00	0.00	0.00	0.00	0.00	0.00	0.00	0.00	0.00	0.00	0.00	0.00	0.00	0.00	0.00	0.00	0.00	0.00	1.00	0.00
PAp3c4(5)	0.00	0.00	0.00	0.00	0.00	0.00	0.00	0.00	0.00	0.00	0.00	0.00	0.00	0.00	0.00	0.00	0.00	0.00	0.00	0.00	0.00	0.00	0.00	0.00	0.00	0.00	0.00	0.00	0.00	0.00	0.00	0.00	0.00	0.20	0.60	0.20

Predicted class (MCR=0.565, SR=43.5%)

Fig. S3 Confusion matrix for hierarchical classification for 3rd level (growth conditions); AO: *Anthoxanthum odoratum* (p1: France, p2: Greece, p3: Finland), FO: *Festuca ovina* (p1: Sweden, p2: Finland, p3: Italy) PA: *Poa alpina* (p1: Sweden; p2: Italy, p3: Norway); c1: 14°C +NU, c2: 14°C -NU, c3: 20°C +NU, c4: 20°C –NU; number of spectra in parenthesis; MCR: misclassification rate, SR: success rate.

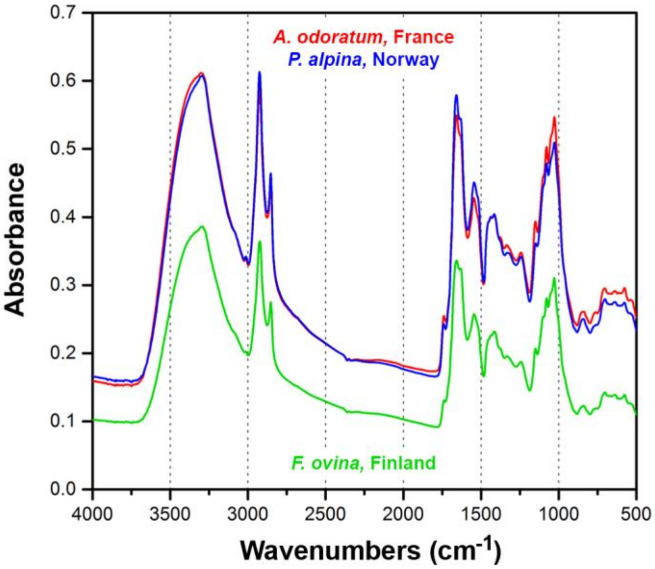

Fig. S4 (Part A) Average spectra of pollen, obtained by averaging of recorded (non pre-processed) spectra belonging to pollen samples collected from plants of the designated population (*Anthoxanthum odoratum* France, *Festuca ovina* Finland, and *Poa alpina* Norway) growing under four different treatments. The plants from the Finish population of *F. ovina* often produced quite small amount of pollen, thus resulting with suboptimal sample quantities for FTIR measurement, and leading to low absorbance values in the corresponding FTIR spectra.

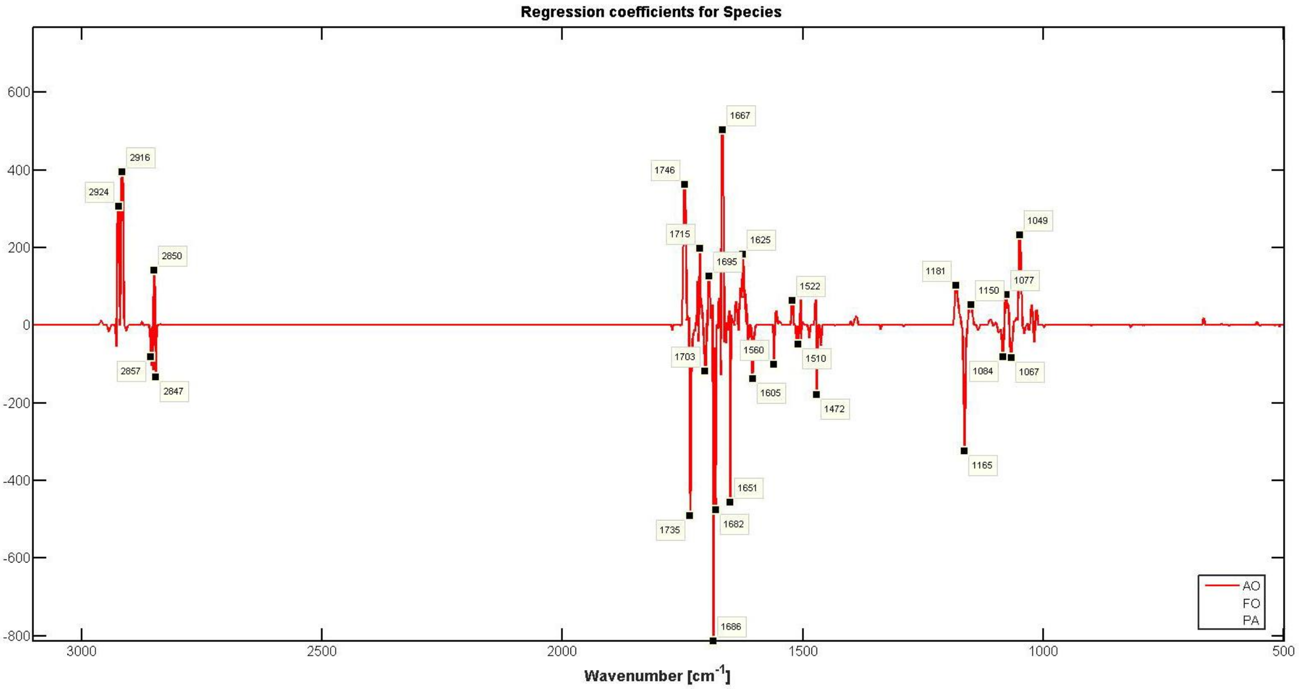

Fig. S4 (Part B) Hierarchical classification for 1st level (species): The regression coefficient for *Anthoxanthum odoratum.*

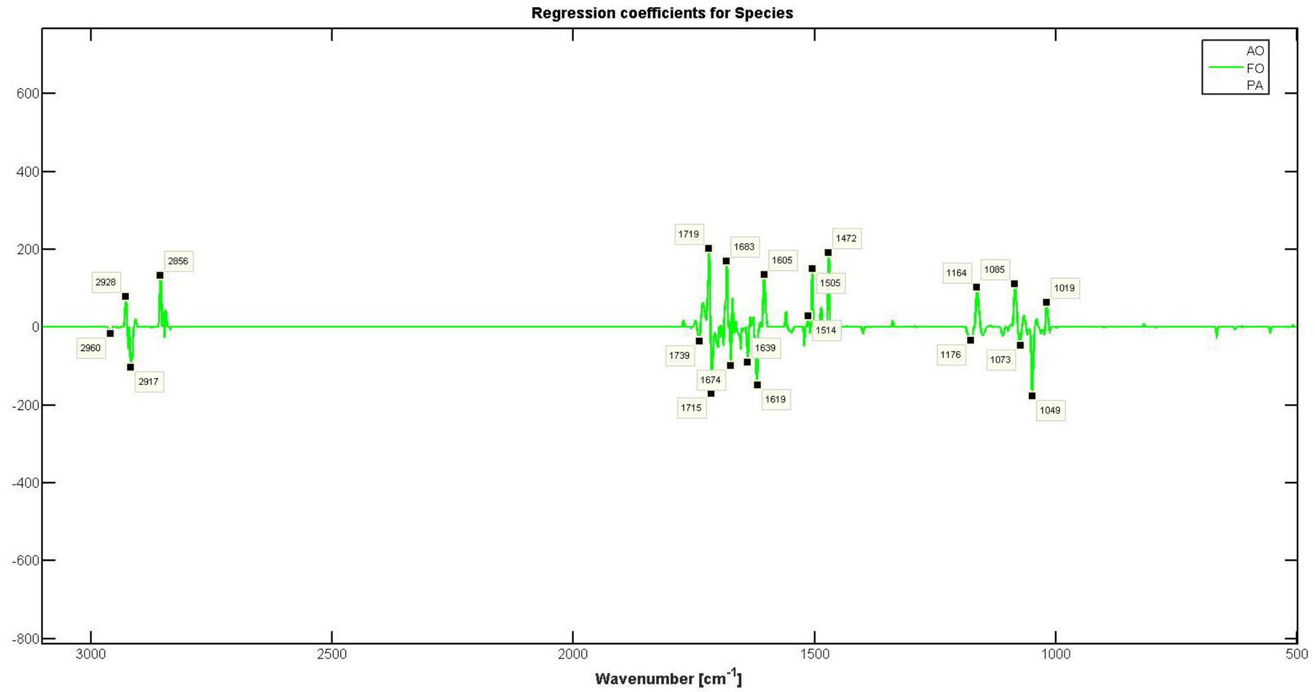

Fig. S4 (Part C) Hierarchical classification for 1st level (species): The regression coefficient for *Festuca ovina.*

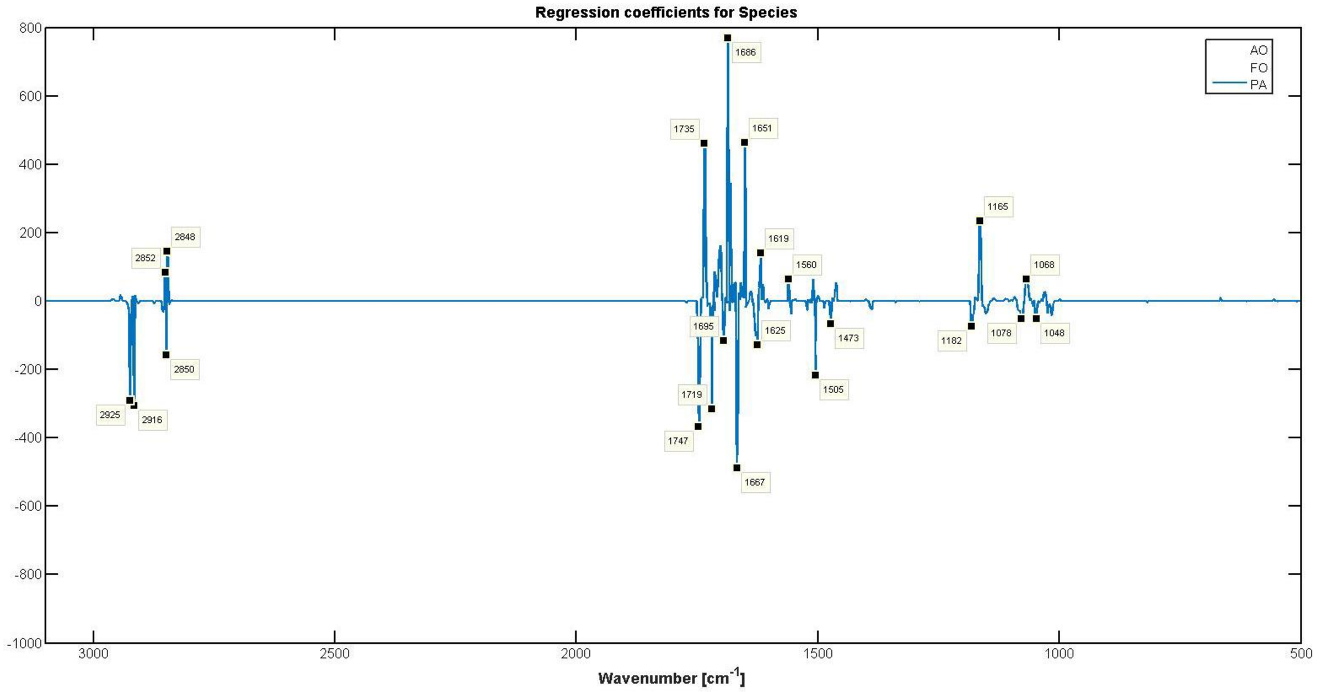

Fig. S4 (Part D) Hierarchical classification for 1st level (species): The regression coefficient for *Poa alpina.*

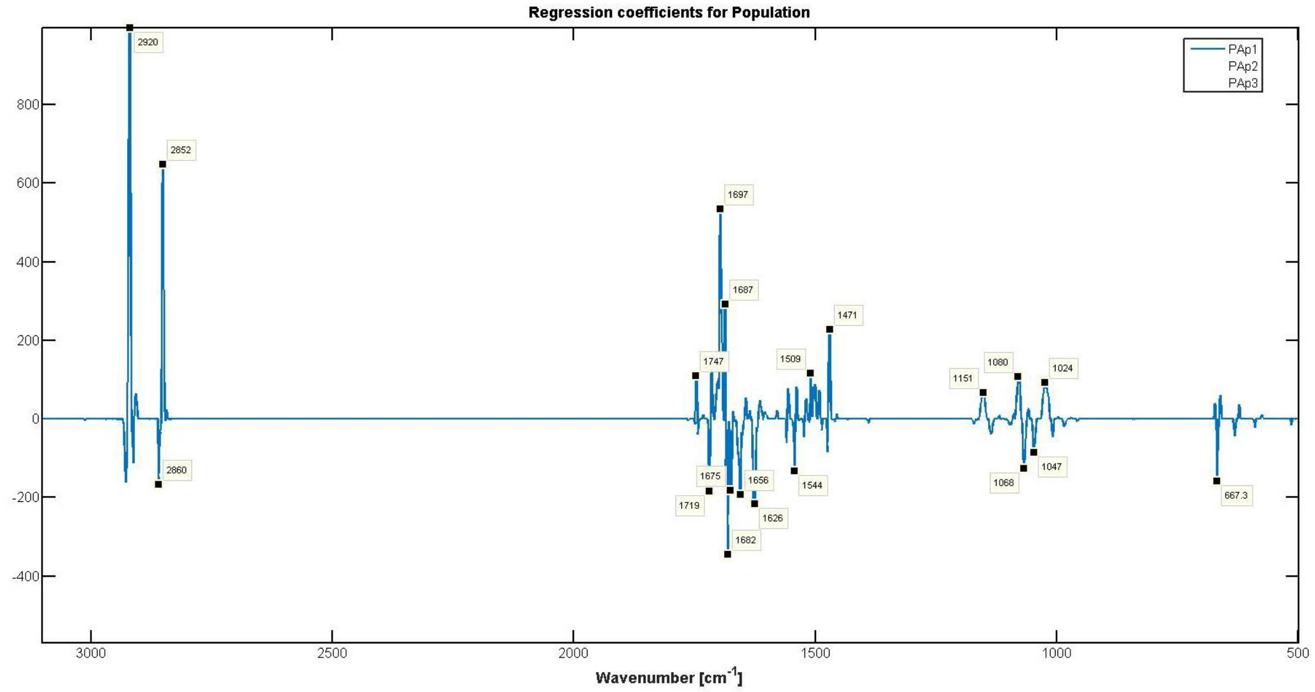

Fig. S5 (Part A) Hierarchical classification for 2nd level (population): The regression coefficient for *Poa alpina*, Sweden.

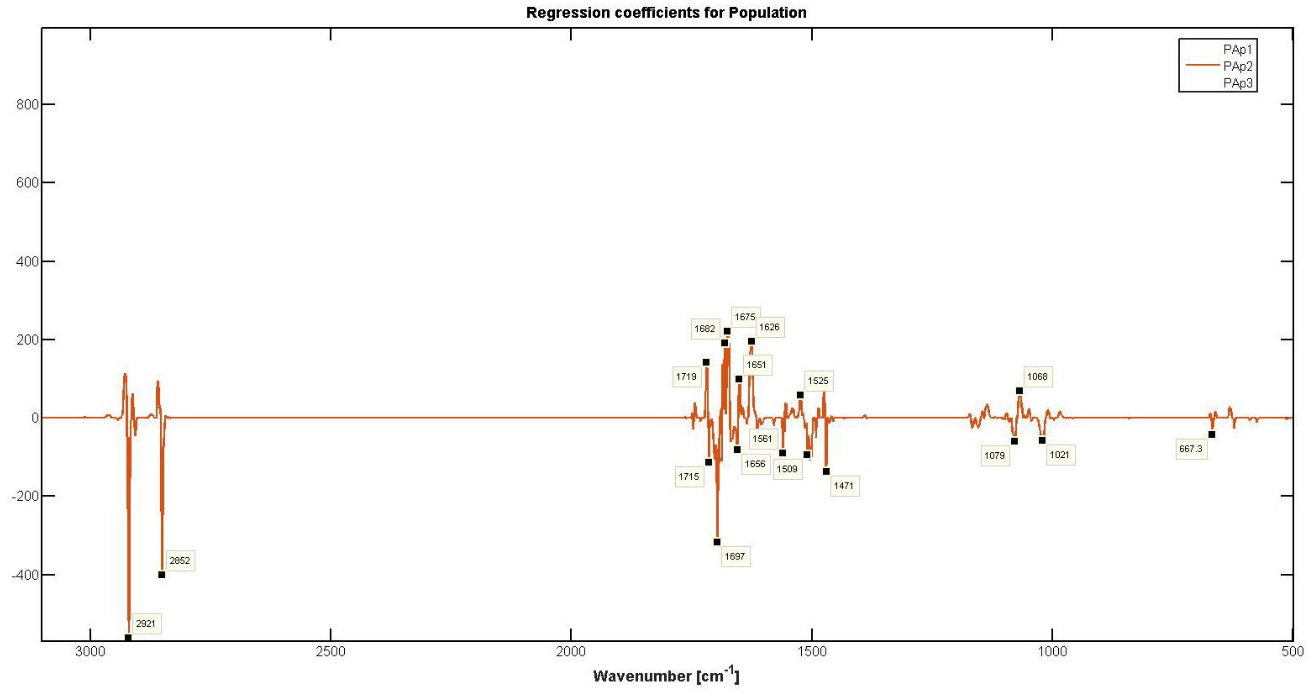

Fig. S5 (Part B) Hierarchical classification for 2nd level (population): The regression coefficient for *Poa alpina*, Italy.

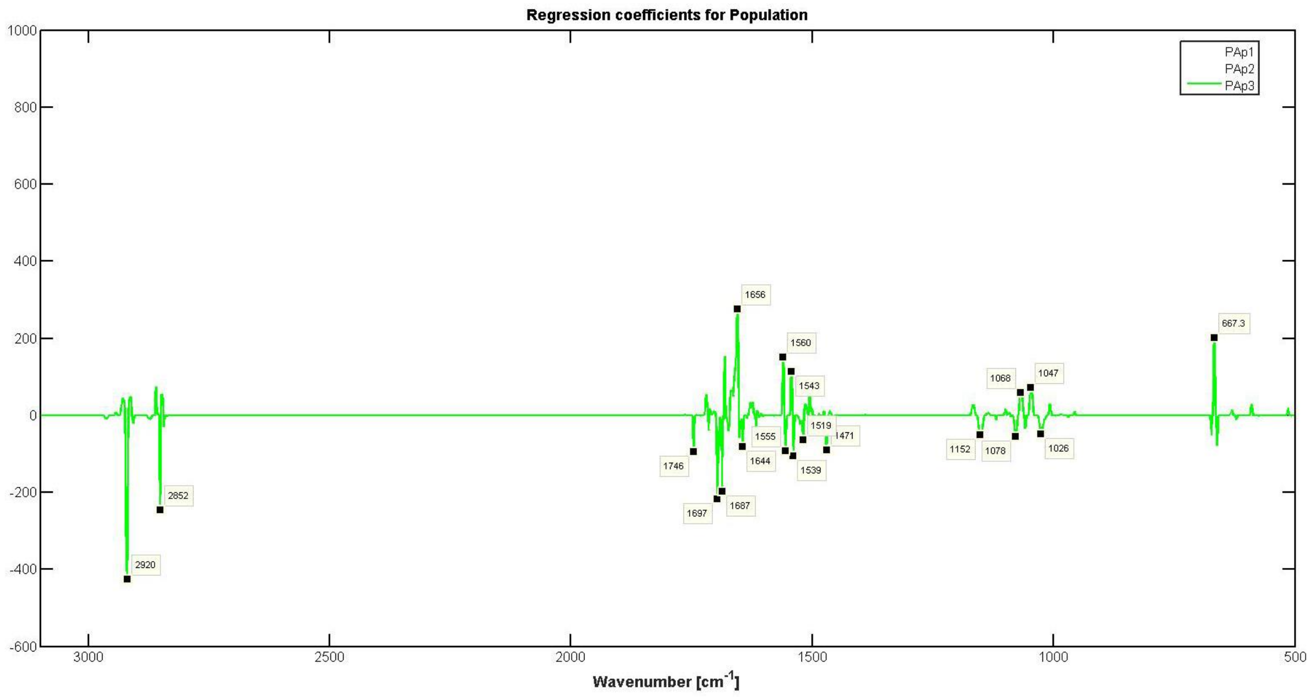

Fig. S5 (Part C) Hierarchical classification for 2nd level (population): The regression coefficient for *Poa alpina*, Norway.

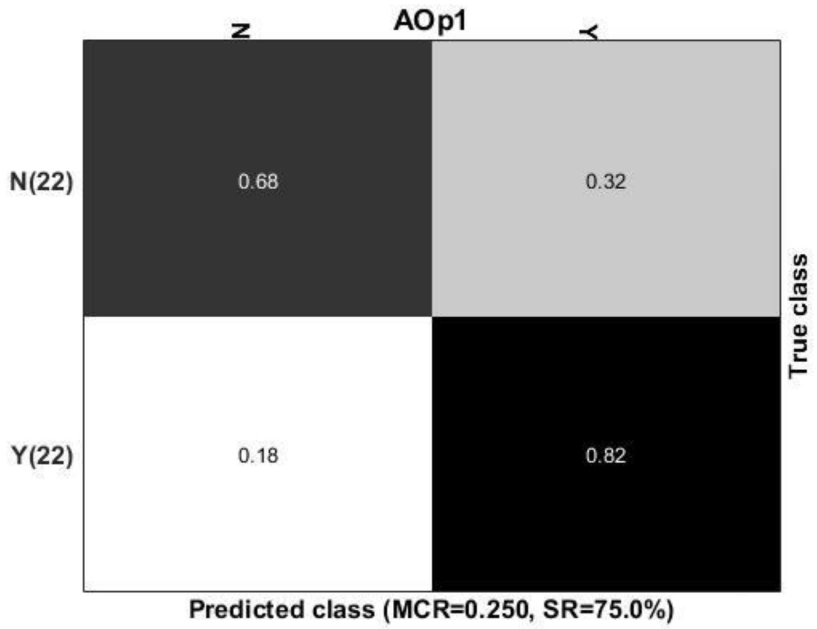

Fig. S6 (Part A) Confusion matrix for classification based on growth conditions; *Anthoxanthum odoratum*, France, N: -NU, Y: +NU; number of spectra in parenthesis; MCR: misclassification rate, SR: success rate.

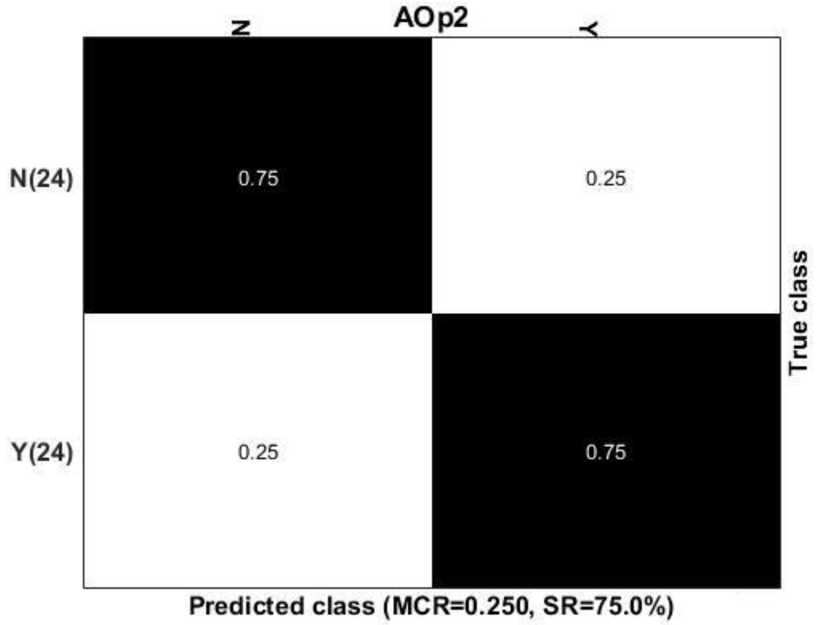

Fig. S6 (Part B) Confusion matrix for classification based on growth conditions; *Anthoxanthum odoratum*, Greece, N: -NU, Y: +NU; number of spectra in parenthesis; MCR: misclassification rate, SR: success rate.

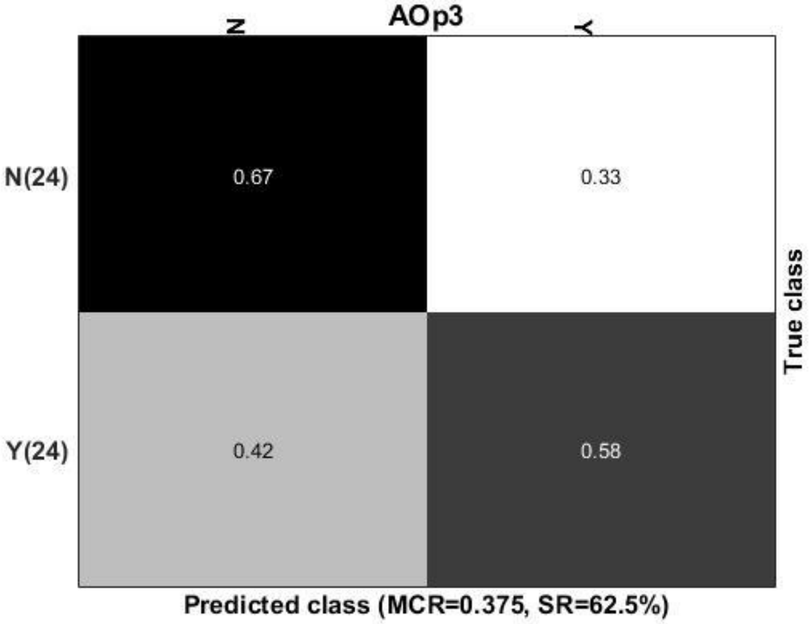

Fig. S6 (Part C) Confusion matrix for classification based on growth conditions; *Anthoxanthum odoratum*, Finland, N: -NU, Y: +NU; number of spectra in parenthesis; MCR: misclassification rate, SR: success rate.

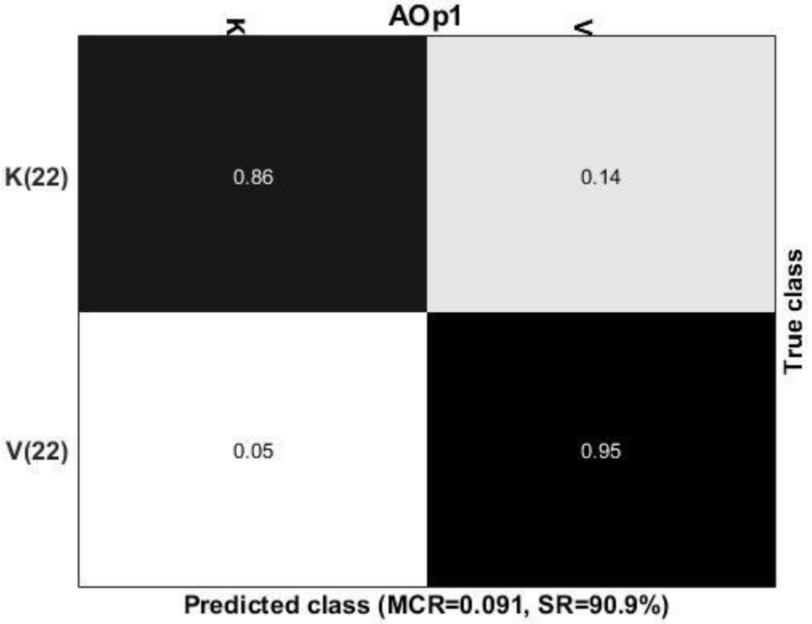

Fig. S6 (Part D) Confusion matrix for classification based on growth conditions; *Anthoxanthum odoratum*, France, K: 14 °C, V: 20 °C; number of spectra in parenthesis; MCR: misclassification rate, SR: success rate.

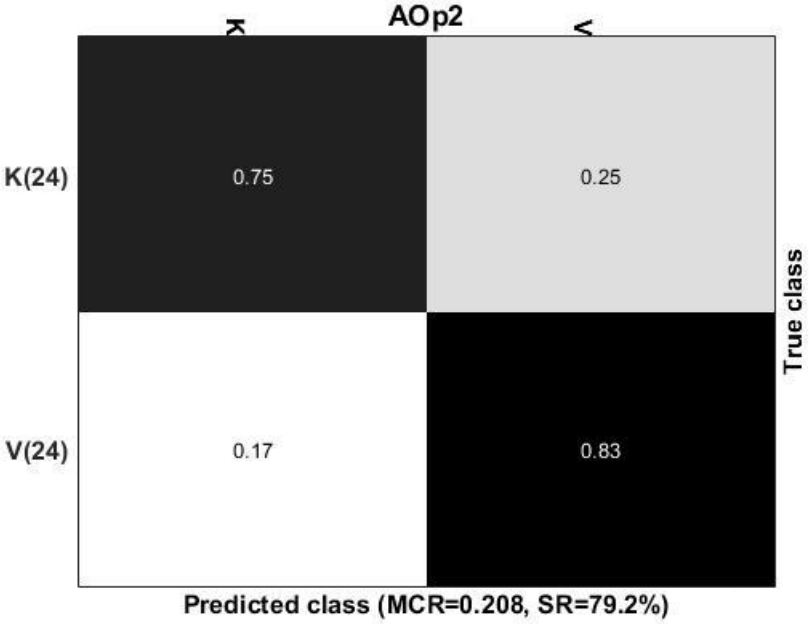

Fig. S6 (Part E) Confusion matrix for classification based on growth conditions; *Anthoxanthum odoratum*, Greece, K: 14 °C, V: 20 °C; number of spectra in parenthesis; MCR: misclassification rate, SR: success rate.

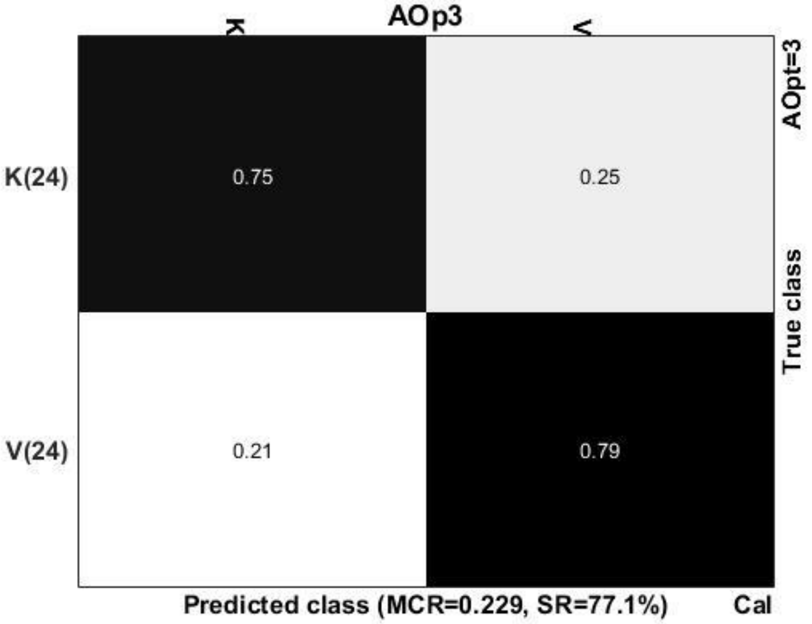

Fig. S6 (Part F) Confusion matrix for classification based on growth conditions; *Anthoxanthum odoratum*, Finland, K: 14 °C, V: 20 °C; number of spectra in parenthesis; MCR: misclassification rate, SR: success rate.

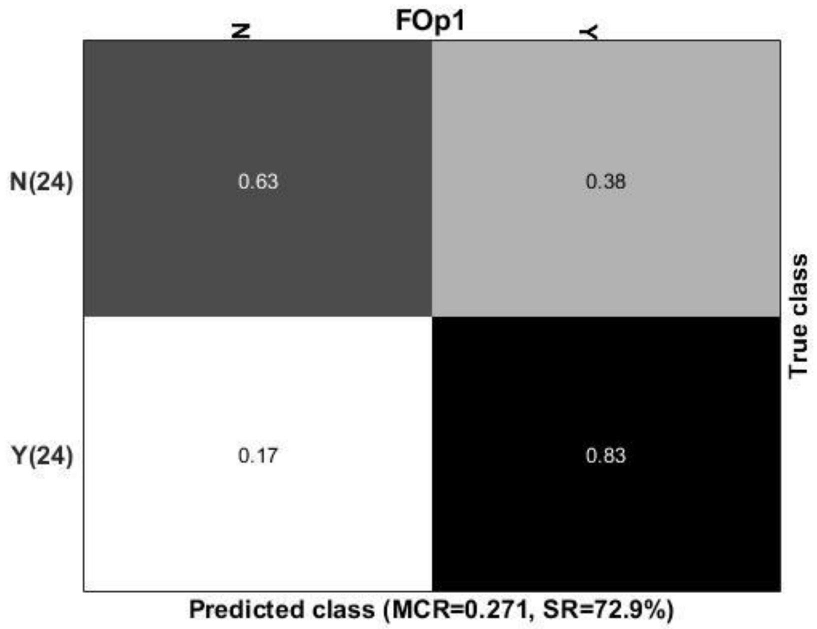

Fig. S7 (Part A) Confusion matrix for classification based on growth conditions; *Festuca ovina*, Sweden, N: -NU, Y: +NU; number of spectra in parenthesis; MCR: misclassification rate, SR: success rate.

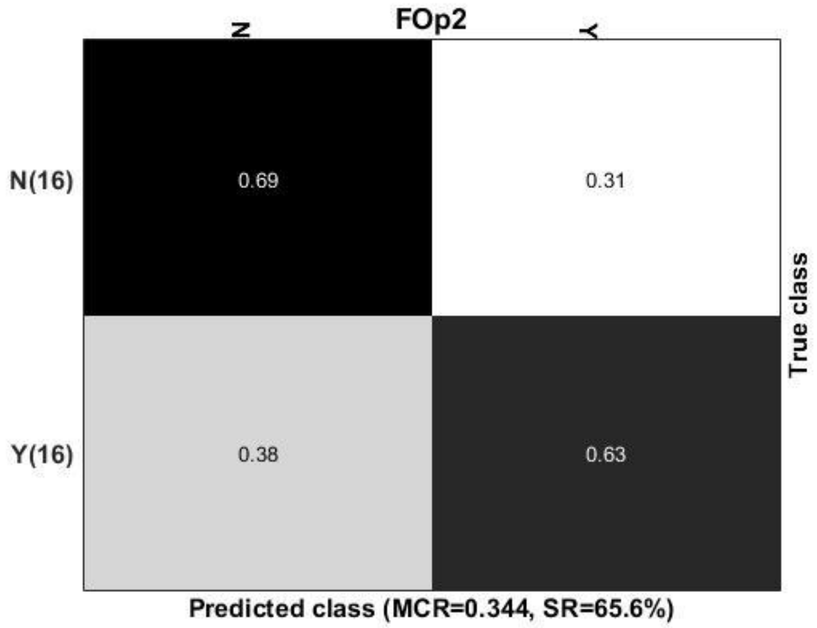

Fig. S7 (Part B) Confusion matrix for classification based on growth conditions; *Festuca ovina*, Finland, N: -NU, Y: +NU; number of spectra in parenthesis; MCR: misclassification rate, SR: success rate.

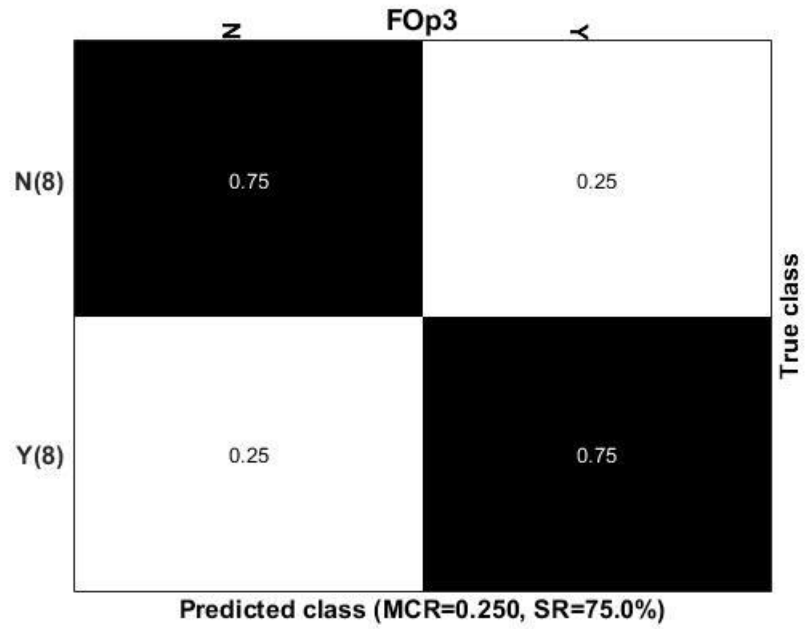

Fig. S7 (Part C) Confusion matrix for classification based on growth conditions; *Festuca ovina*, Italy, N: -NU, Y: +NU; number of spectra in parenthesis; MCR: misclassification rate, SR: success rate.

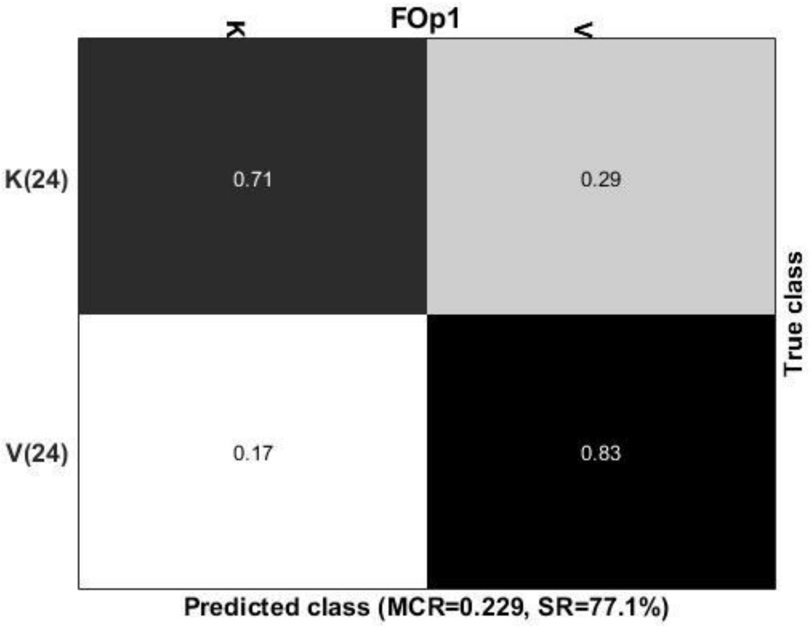

Fig. S7 (Part D) Confusion matrix for classification based on growth conditions; *Festuca ovina*, Sweden, K: 14 °C, V: 20 °C; number of spectra in parenthesis; MCR: misclassification rate, SR: success rate.

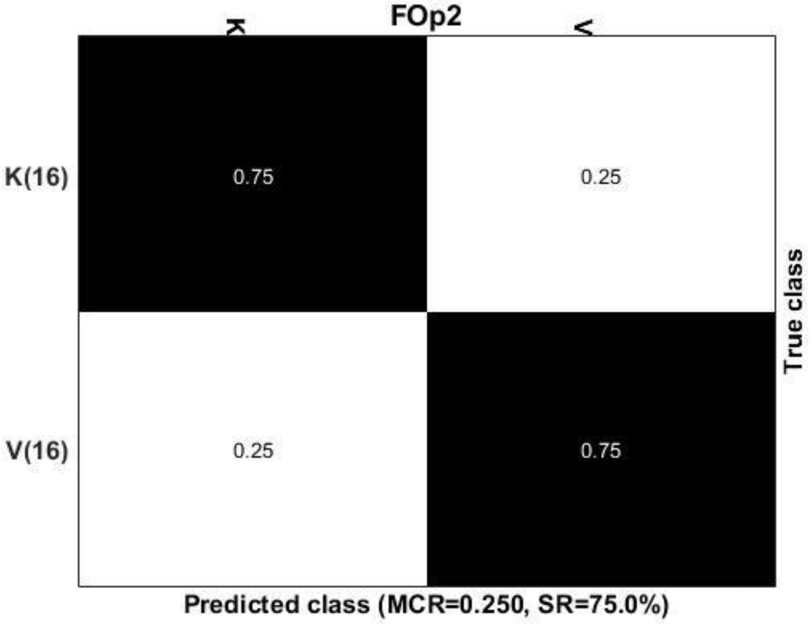

Fig. S7 (Part E) Confusion matrix for classification based on growth conditions; *Festuca ovina*, Finland, K: 14 °C, V: 20 °C; number of spectra in parenthesis; MCR: misclassification rate, SR: success rate.

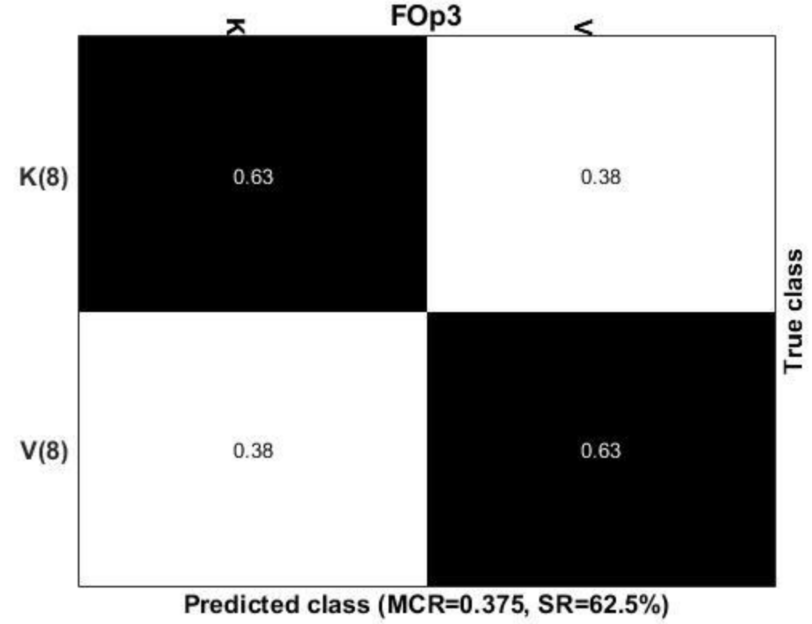

Fig. S7 (Part F) Confusion matrix for classification based on growth conditions; *Festuca ovina*, Italy, K: 14 °C, V: 20 °C; number of spectra in parenthesis; MCR: misclassification rate, SR: success rate.

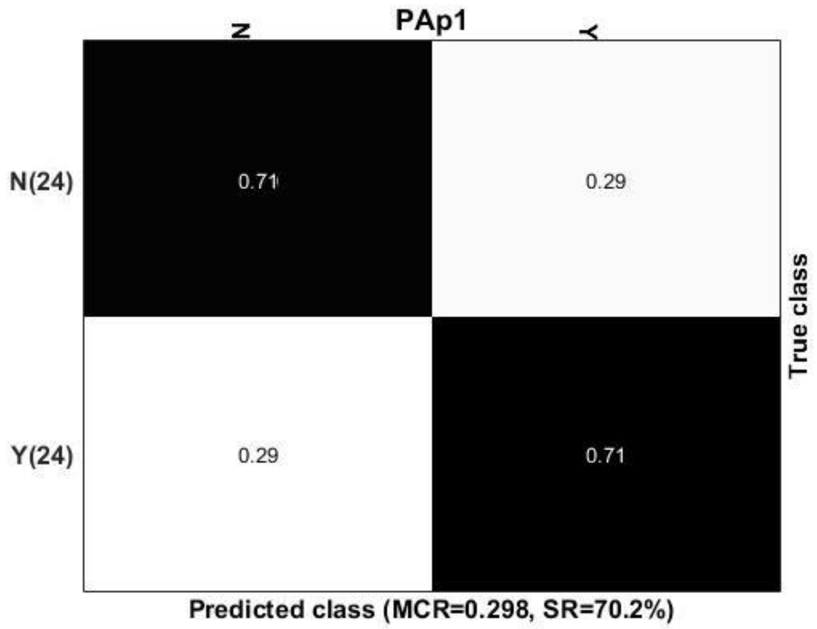

Fig. S8 (Part A) Confusion matrix for classification based on growth conditions; *Poa alpina*, Sweden, N: -NU, Y: +NU; number of spectra in parenthesis; MCR: misclassification rate, SR: success rate.

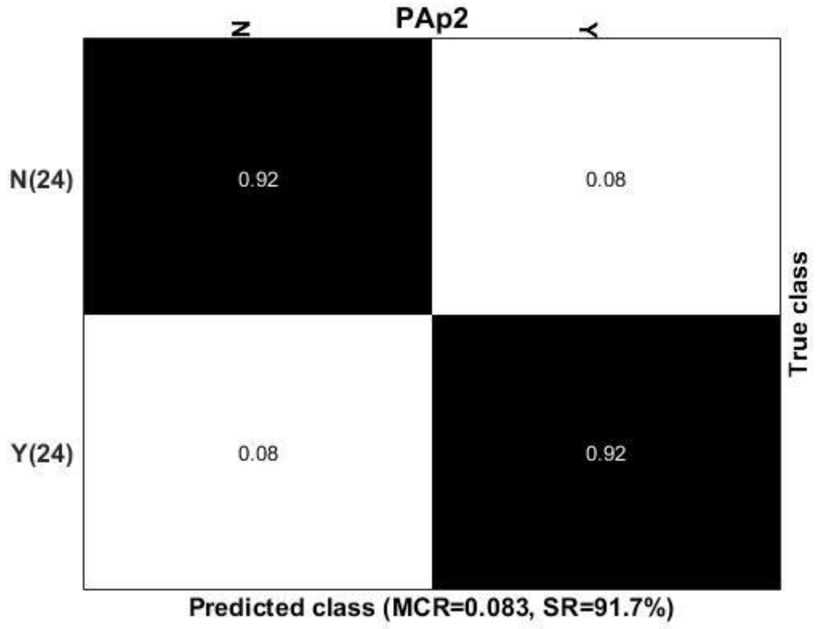

Fig. S8 (Part B) Confusion matrix for classification based on growth conditions; *Poa alpina*, Italy, N: -NU, Y: +NU; number of spectra in parenthesis; MCR: misclassification rate, SR: success rate.

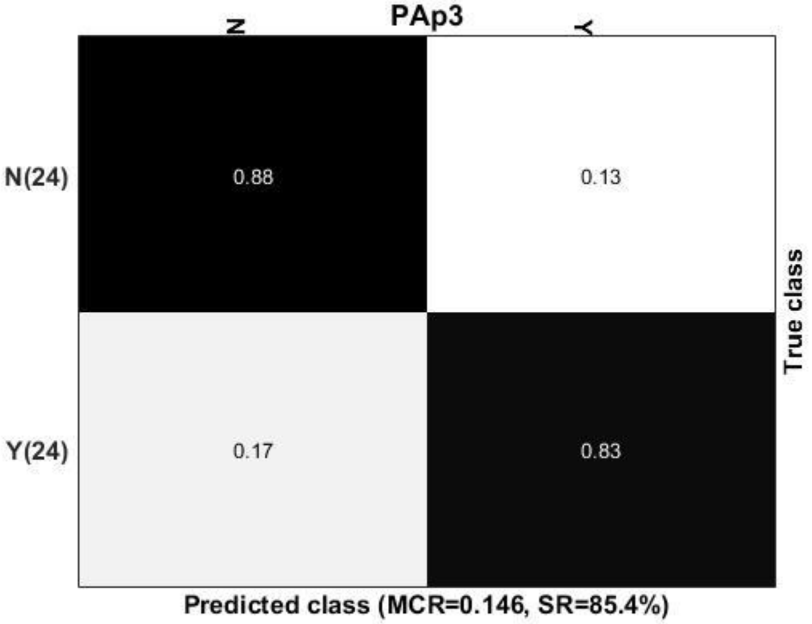

Fig. S8 (Part C) Confusion matrix for classification based on growth conditions; *Poa alpina*, Norway, N: -NU, Y: +NU; number of spectra in parenthesis; MCR: misclassification rate, SR: success rate.

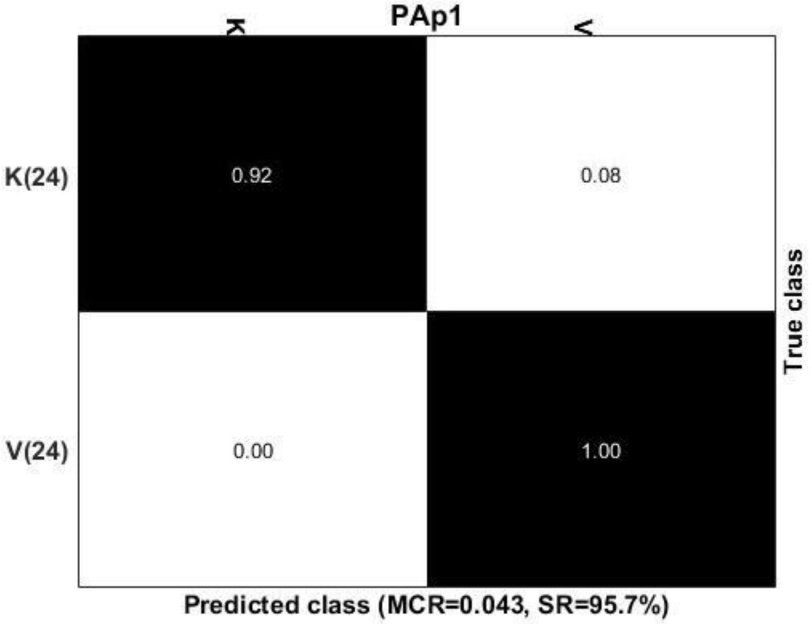

Fig. S8 (Part D) Confusion matrix for classification based on growth conditions; *Poa alpina*, Sweden, K: 14 °C, V: 20 °C; number of spectra in parenthesis; MCR: misclassification rate, SR: success rate.

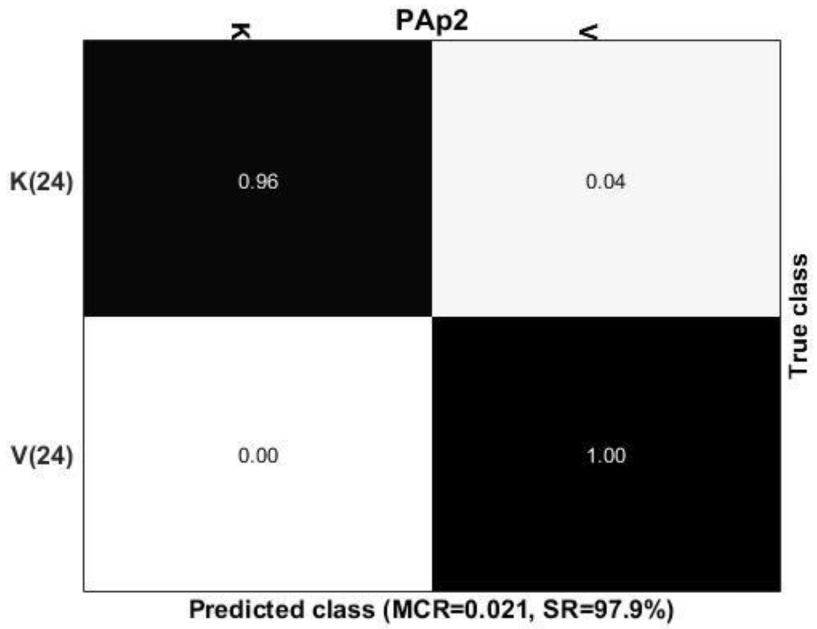

Fig. S8 (Part E) Confusion matrix for classification based on growth conditions; *Poa alpina*, Italy, K: 14 °C, V: 20 °C; number of spectra in parenthesis; MCR: misclassification rate, SR: success rate.

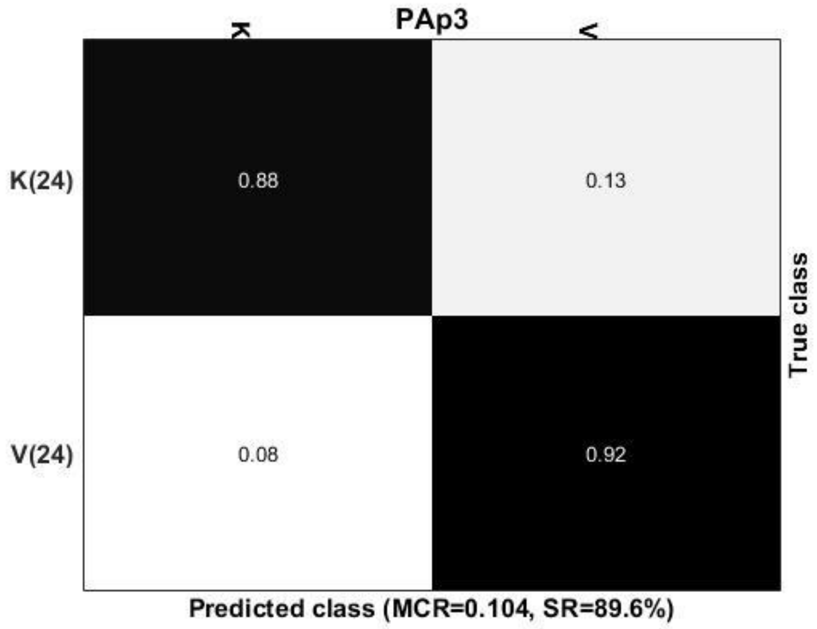

Fig. S8 (Part F) Confusion matrix for classification based on growth conditions; *Poa alpina*, Norway, K: 14 °C, V: 20 °C; number of spectra in parenthesis; MCR: misclassification rate, SR: success rate.

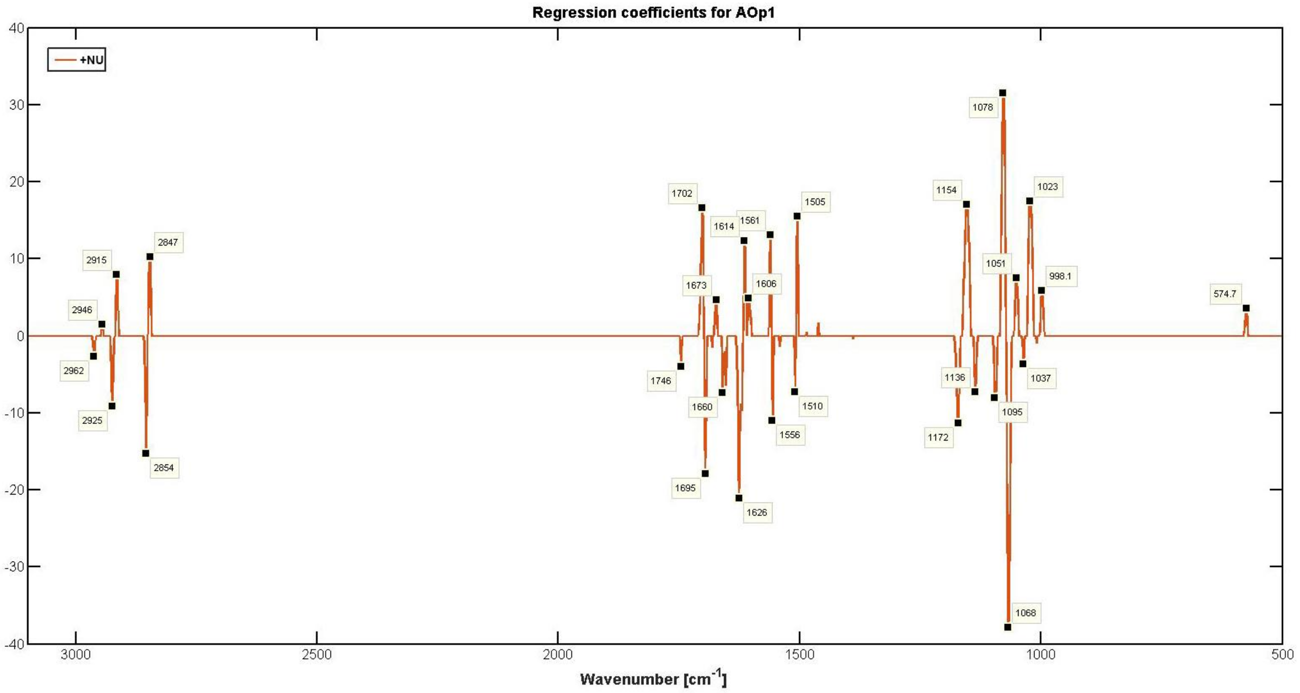

Fig. S9 (Part A) Classification based on growth conditions: The regression coefficient for *Anthoxanthum odoratum*, France, +NU; the regression coefficient for –NU is inverse.

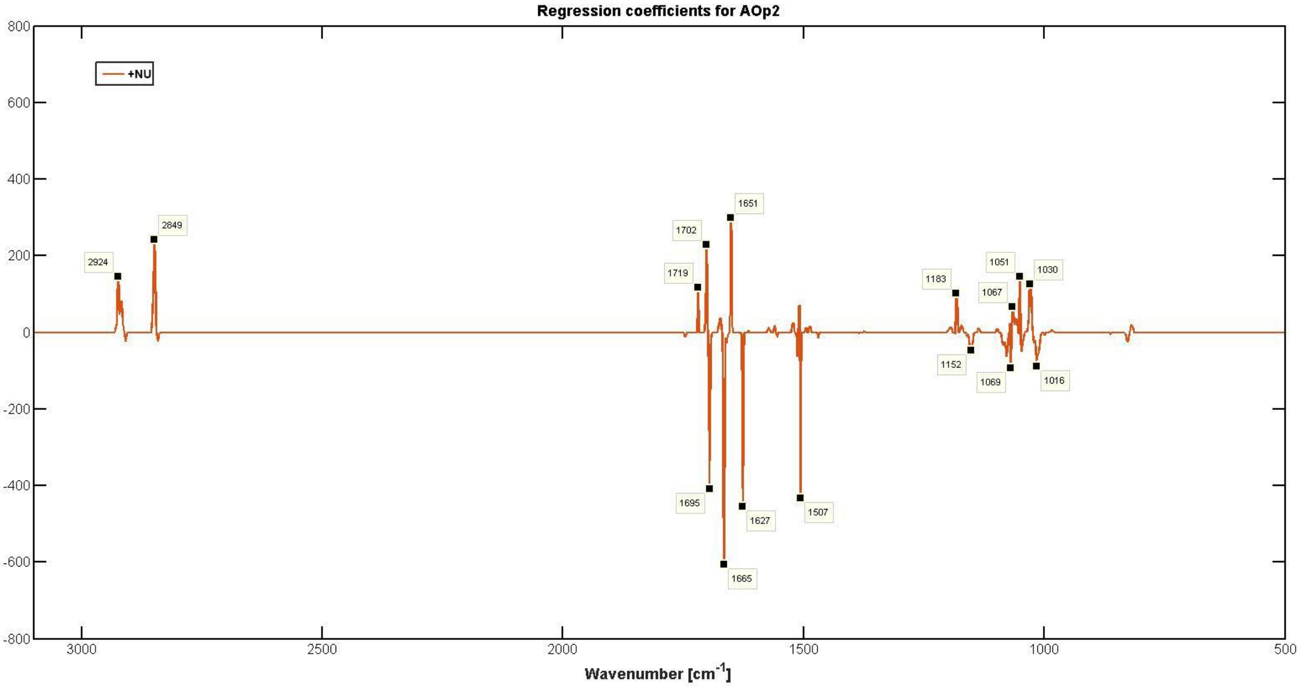

Fig. S9 (Part B) Classification based on growth conditions: The regression coefficient for *Anthoxanthum odoratum*, Greece, +NU; the regression coefficient for –NU is inverse.

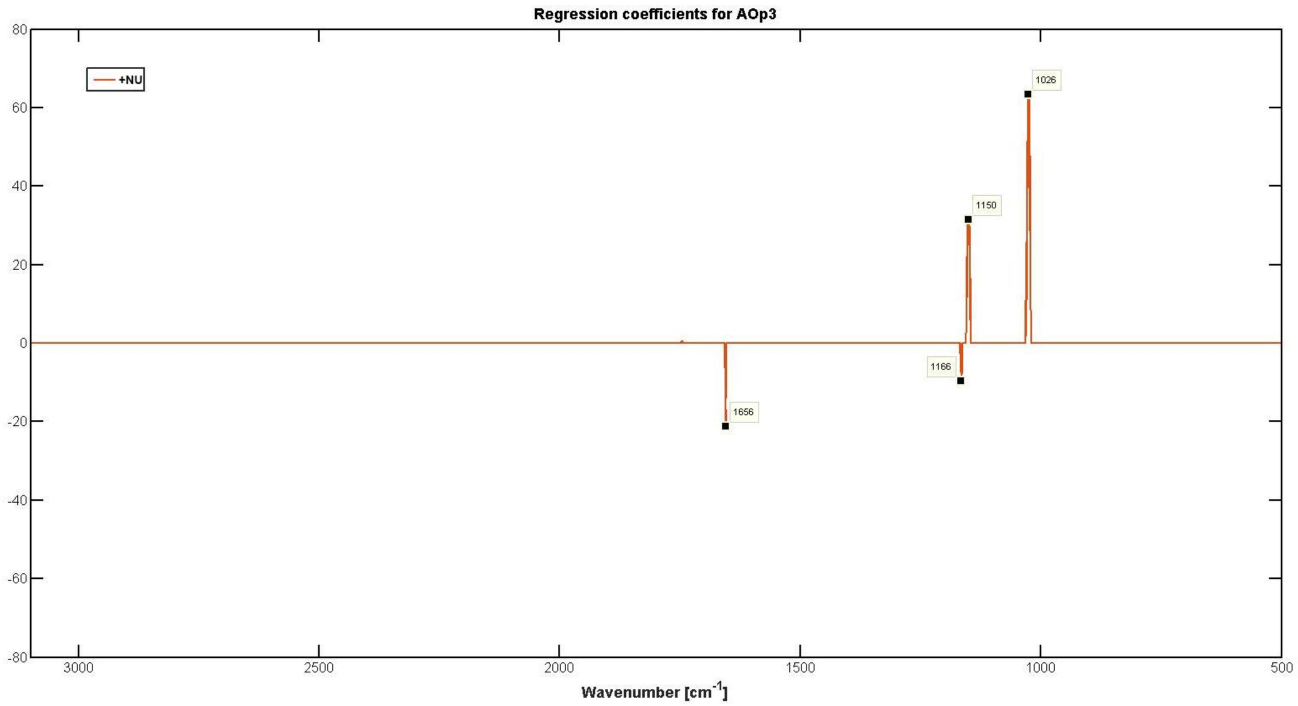

Fig. S9 (Part C) Classification based on growth conditions: The regression coefficient for *Anthoxanthum odoratum*, Finland, +NU; the regression coefficient for –NU is inverse.

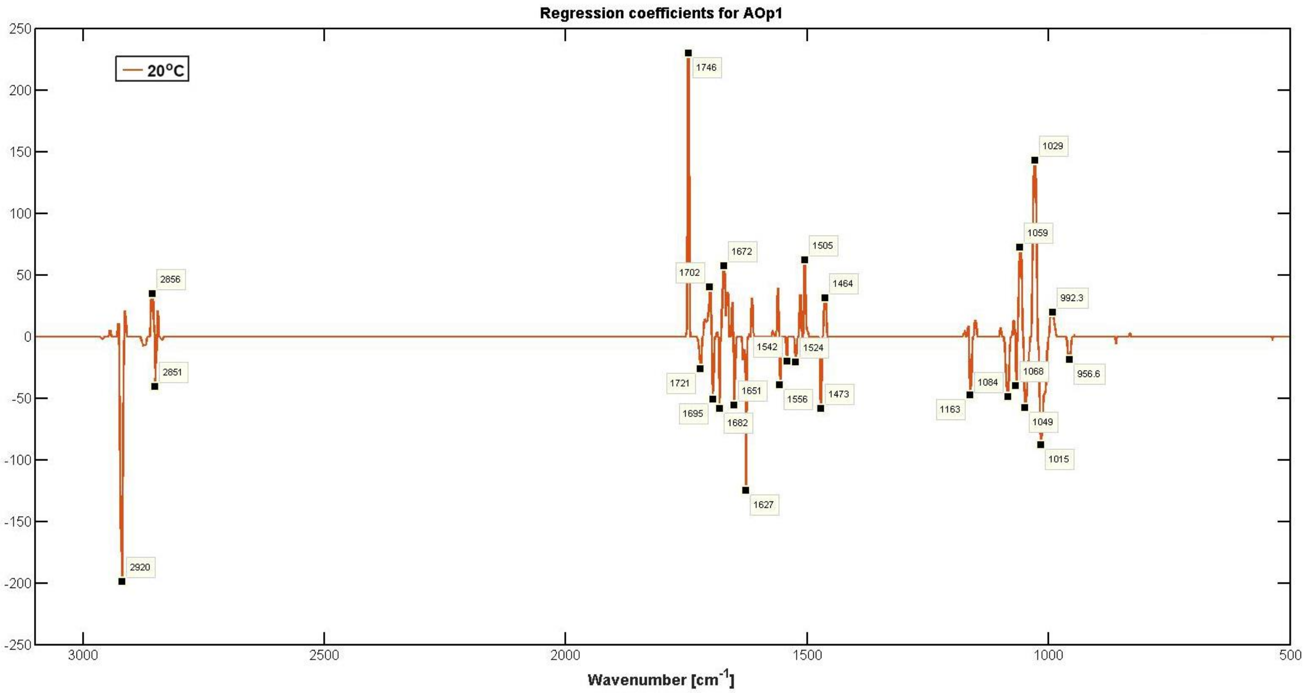

Fig. S9 (Part D) Classification based on growth conditions: The regression coefficient for *Anthoxanthum odoratum*, France, 20°C; the regression coefficient for 14°C is inverse.

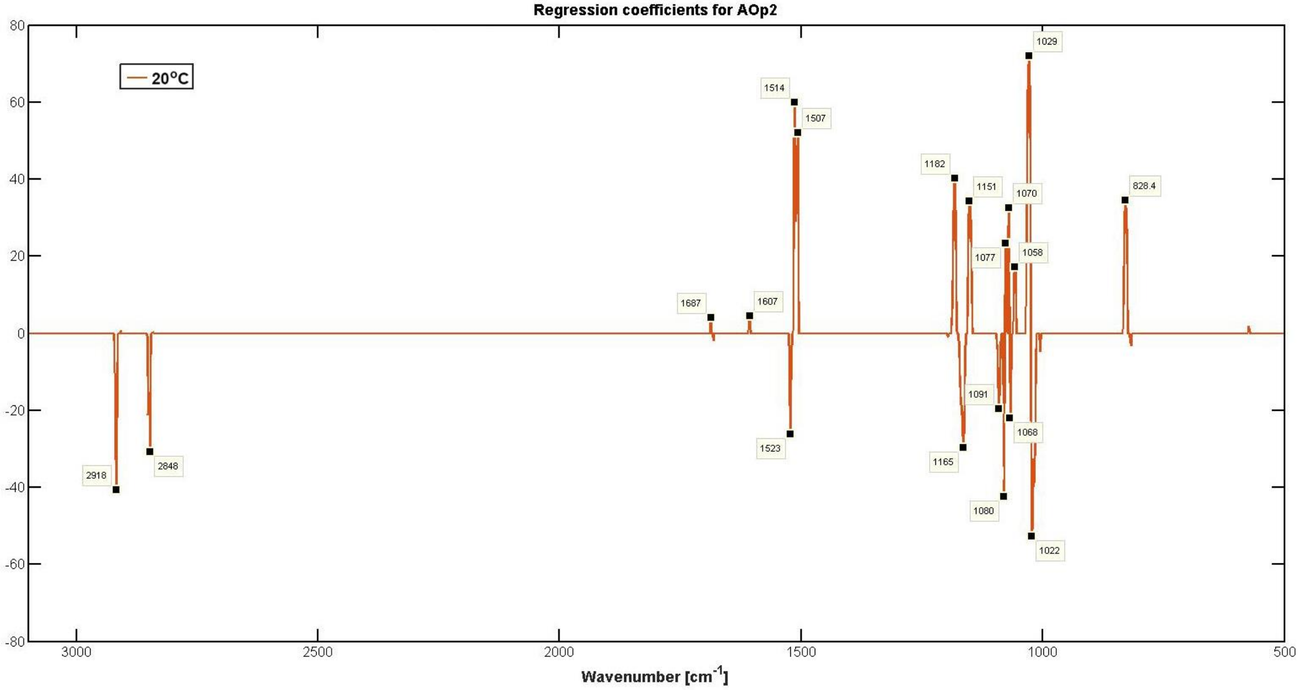

Fig. S9 (Part E) Classification based on growth conditions: The regression coefficient for *Anthoxanthum odoratum*, Greece, 20°C; the regression coefficient for 14°C is inverse.

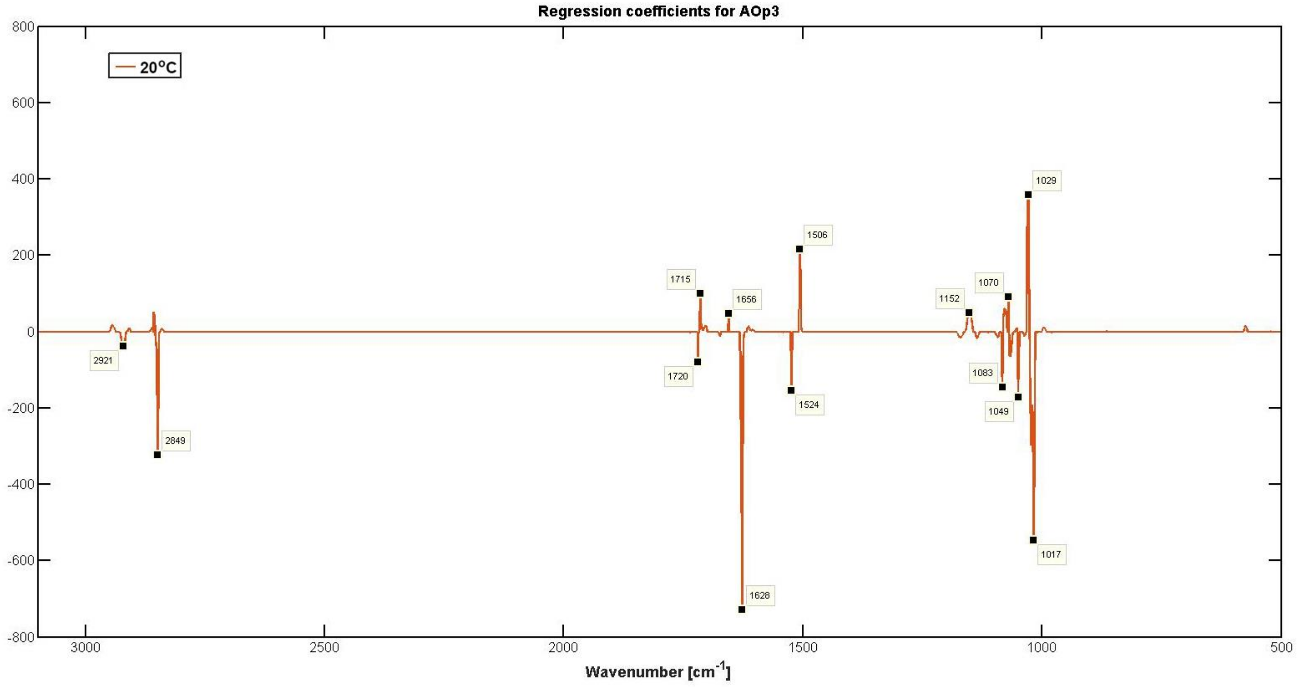

Fig. S9 (Part F) Classification based on growth conditions: The regression coefficient for *Anthoxanthum odoratum*, Finland, 20°C; the regression coefficient for 14°C is inverse.

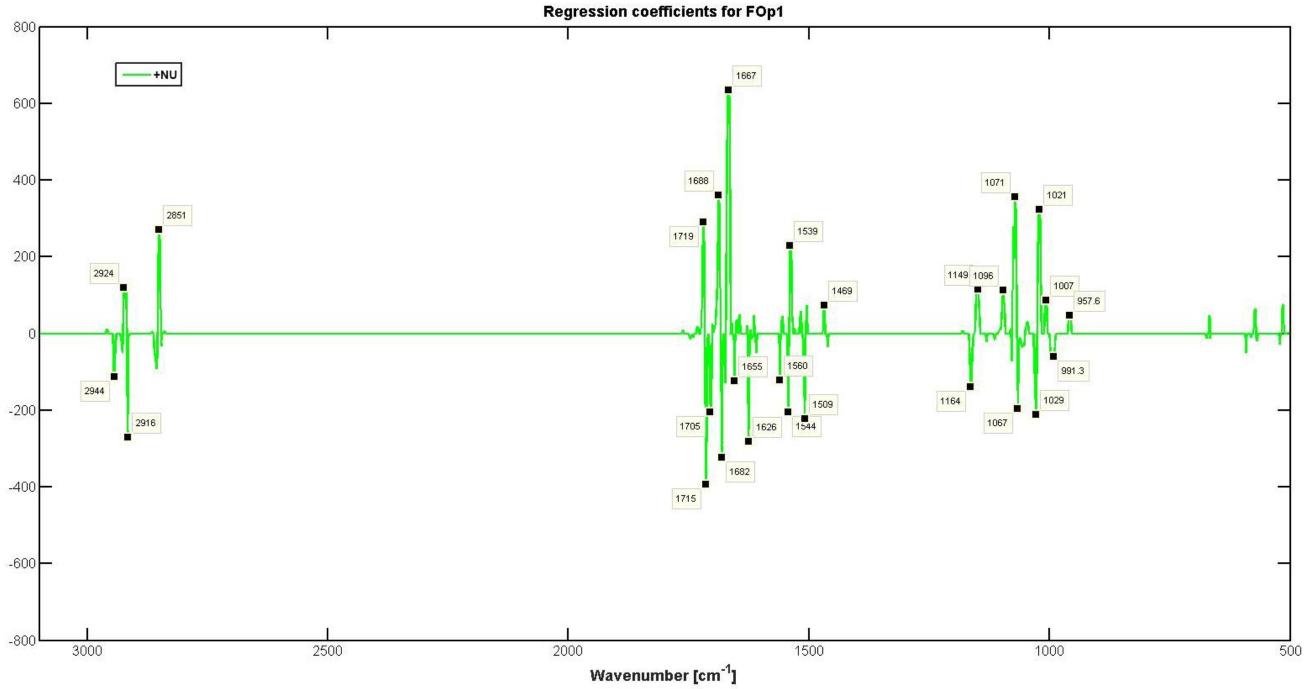

Fig. S10 (Part A) Classification based on growth conditions: The regression coefficient for *Festuca ovina*, Sweden, +NU; the regression coefficient for –NU is inverse.

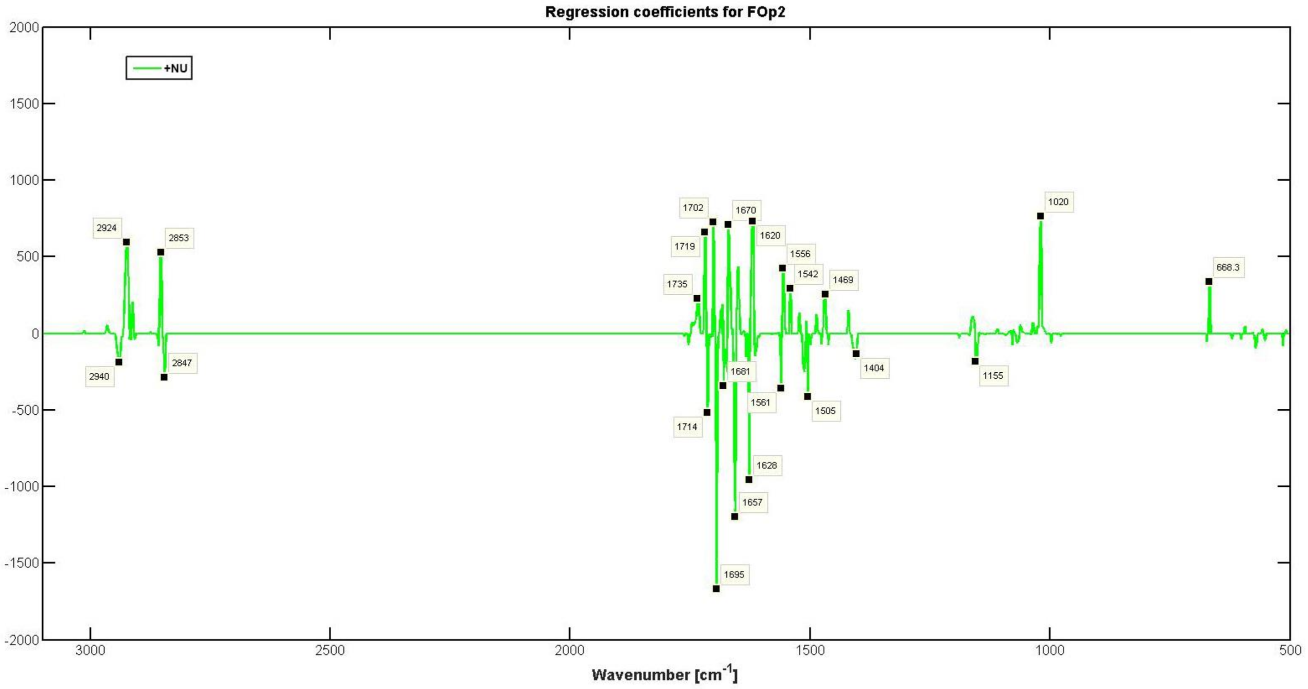

Fig. S10 (Part B) Classification based on growth conditions: The regression coefficient for *Festuca ovina*, Finland, +NU; the regression coefficient for –NU is inverse.

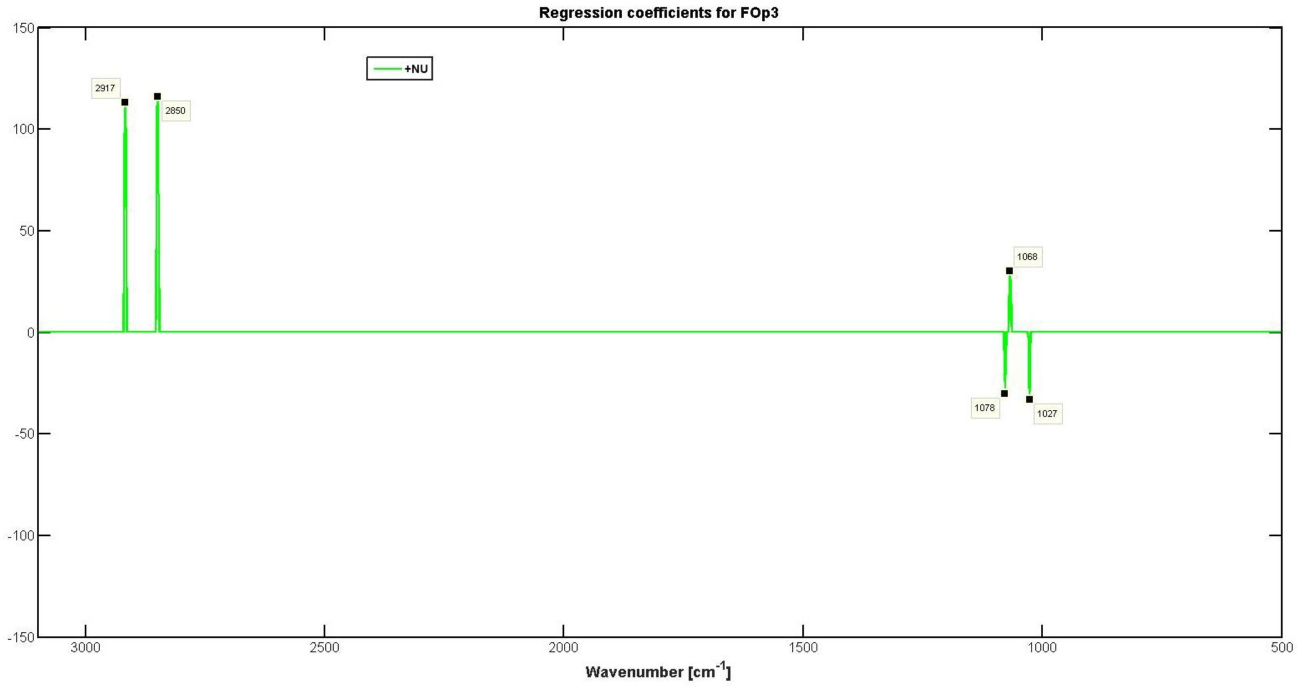

Fig. S10 (Part C) Classification based on growth conditions: The regression coefficient for *Festuca ovina*, Italy, +NU; the regression coefficient for –NU is inverse.

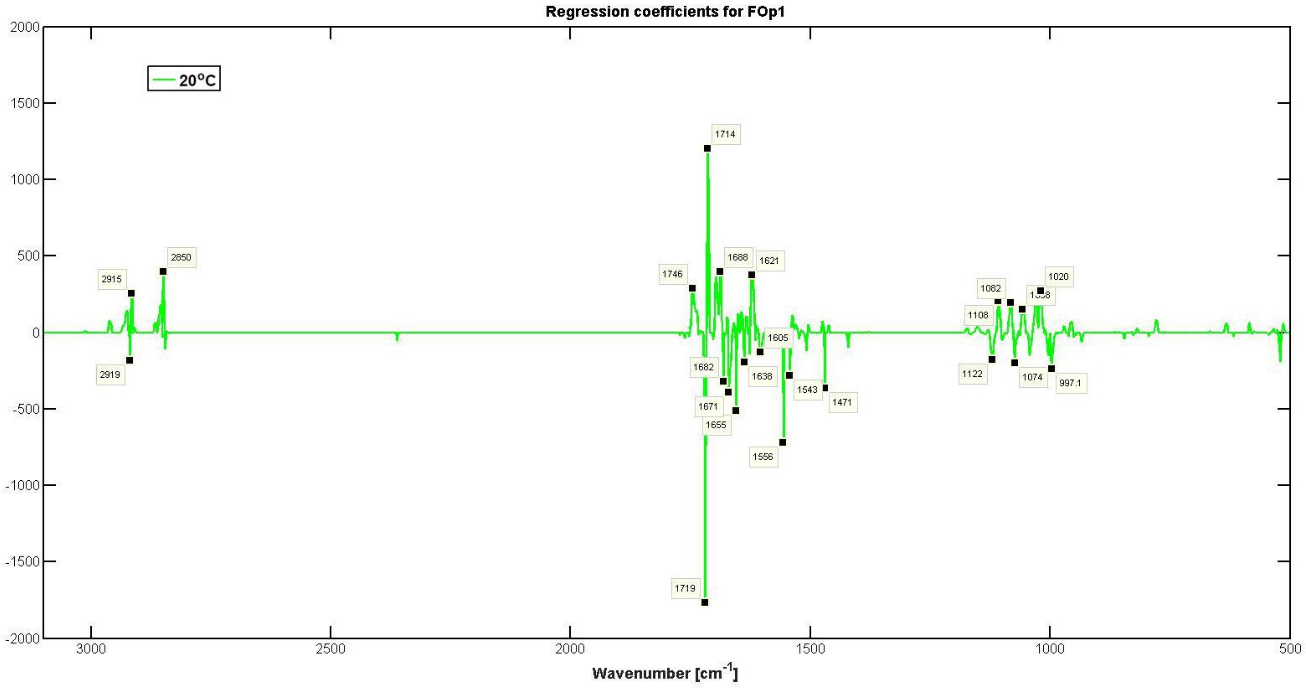

Fig. S10 (Part D) Classification based on growth conditions: The regression coefficient for *Festuca ovina*, Sweden, 20°C; the regression coefficient for 14°C is inverse.

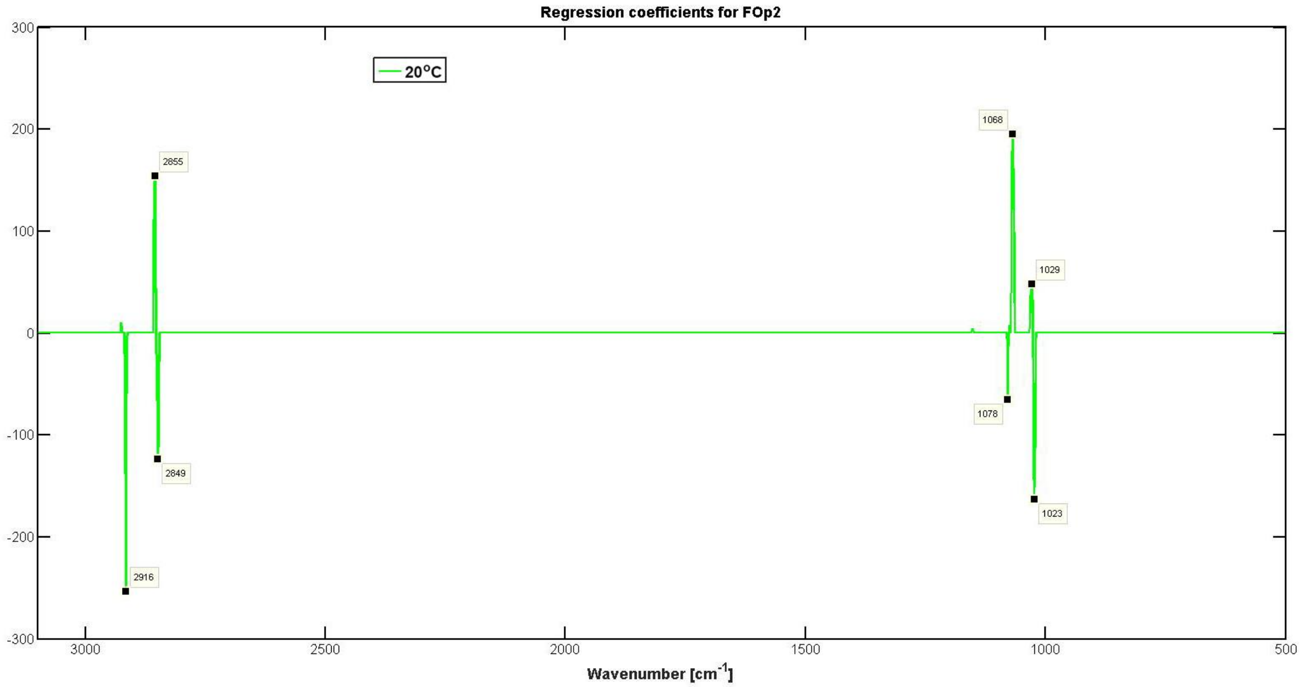

Fig. S10 (Part E) Classification based on growth conditions: The regression coefficient for *Festuca ovina*, Finland, 20°C; the regression coefficient for 14°C is inverse.

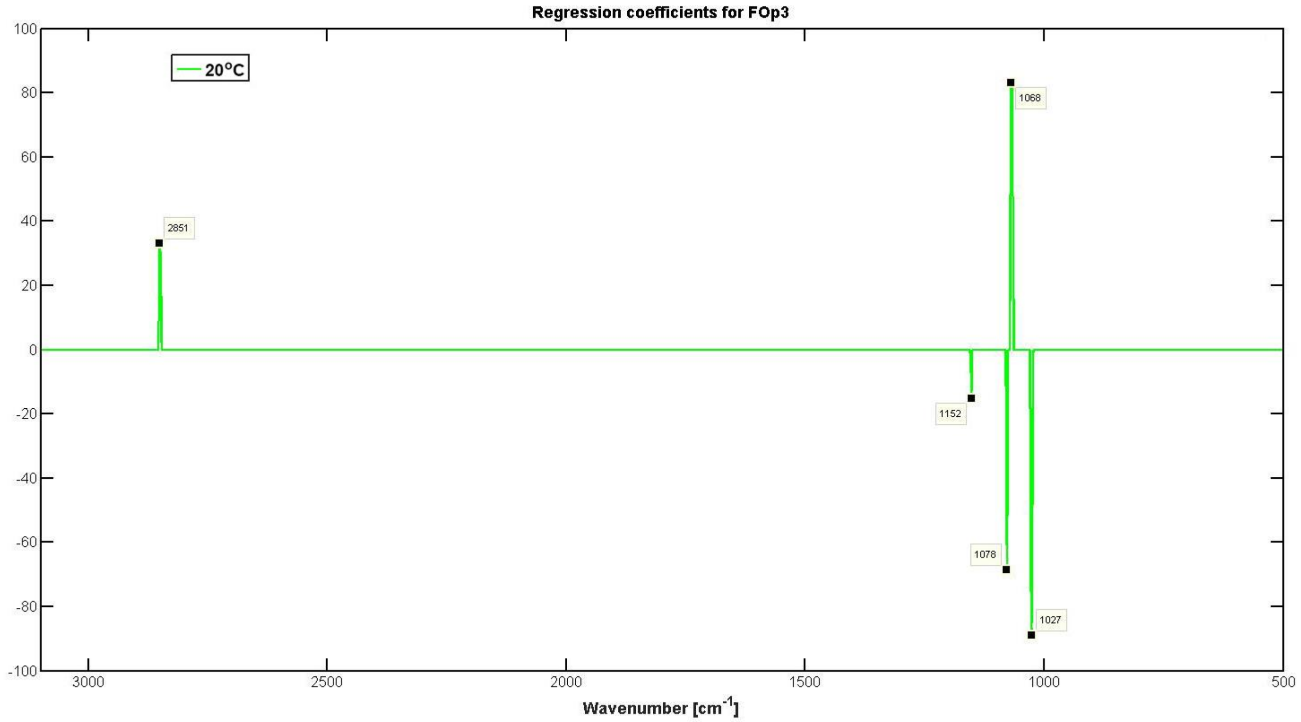

Fig. S10 (Part F) Classification based on growth conditions: The regression coefficient for *Festuca ovina*, Italy, 20°C; the regression coefficient for 14°C is inverse.

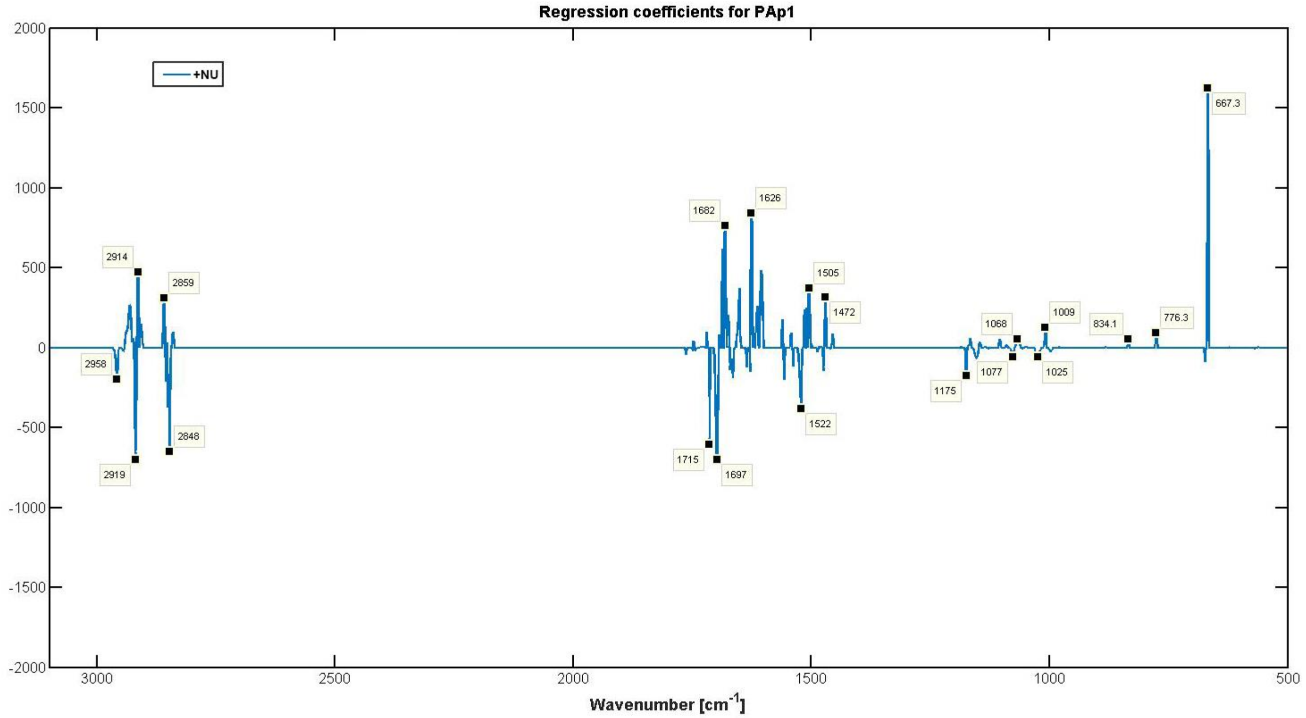

Fig. S11 (Part A) Classification based on growth conditions: The regression coefficient for *Poa alpina*, Sweden, +NU; The regression coefficient for –NU is inverse.

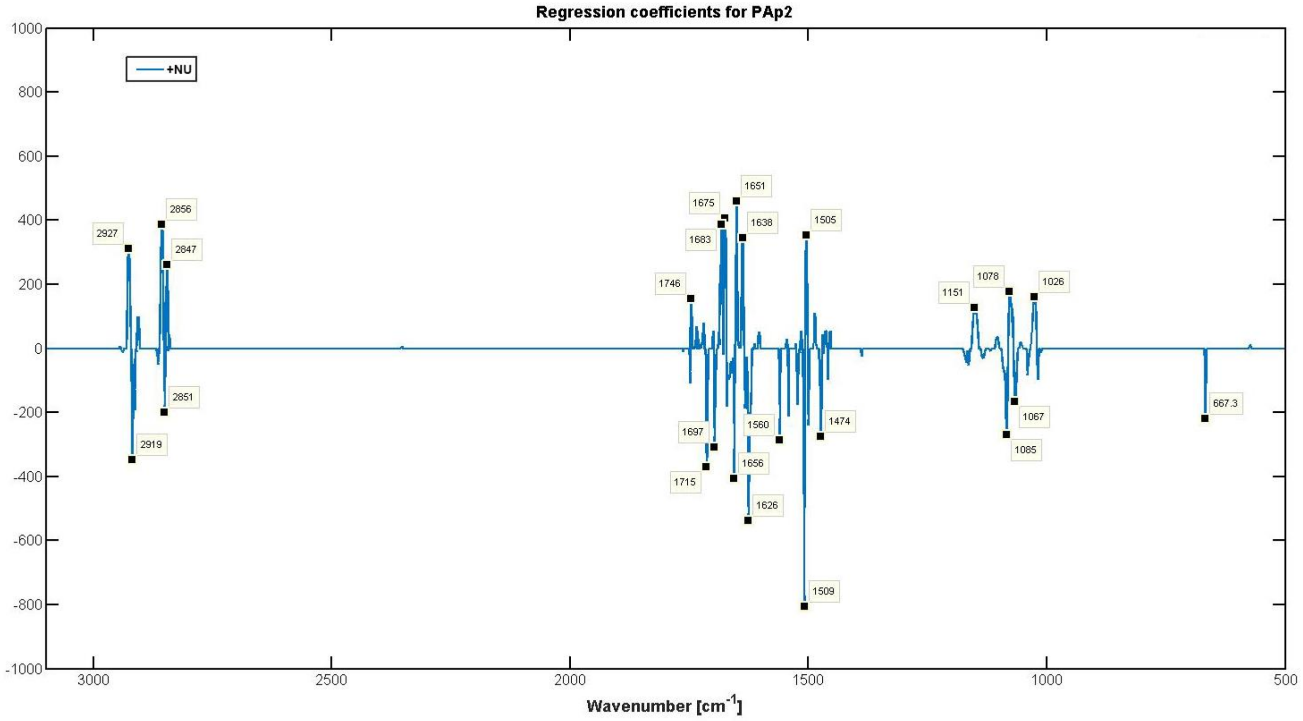

Fig. S11 (Part B) Classification based on growth conditions: The regression coefficient for *Poa alpina*, Italy, +NU; The regression coefficient for –NU is inverse.

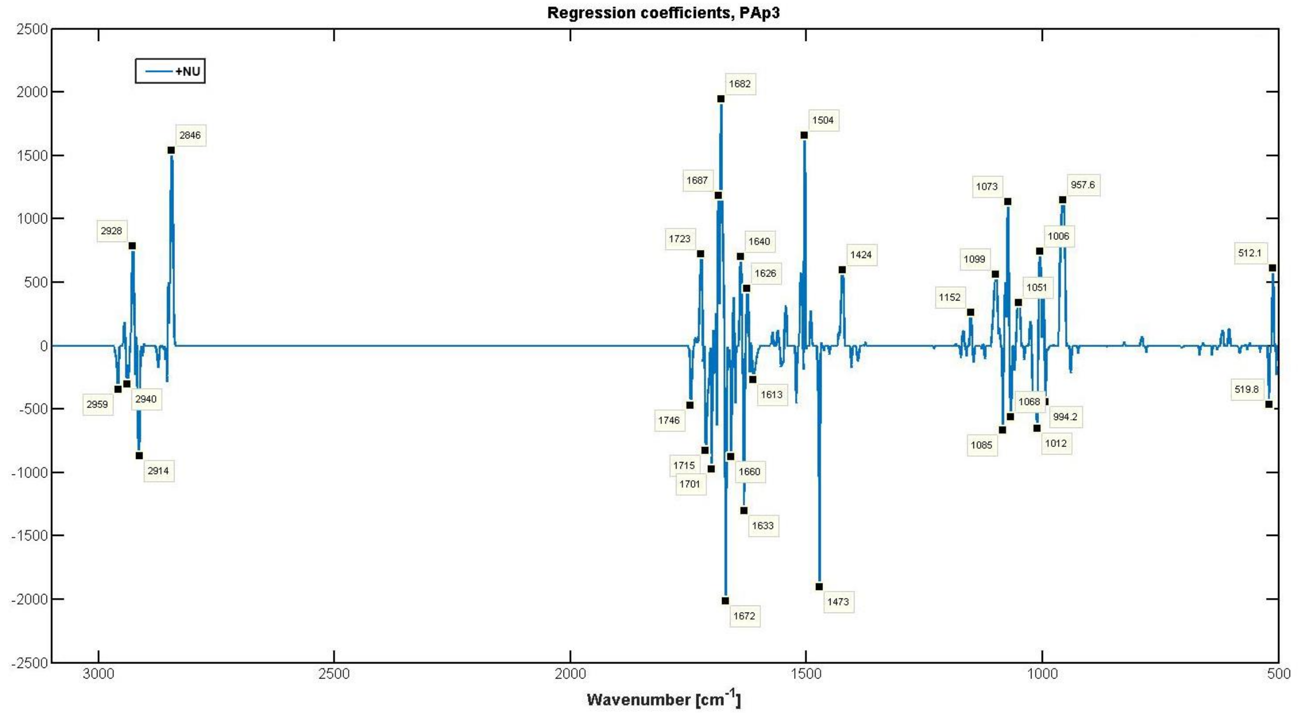

Fig. S11 (Part C) Classification based on growth conditions: The regression coefficient for *Poa alpina*, Norway, +NU; The regression coefficient for –NU is inverse.

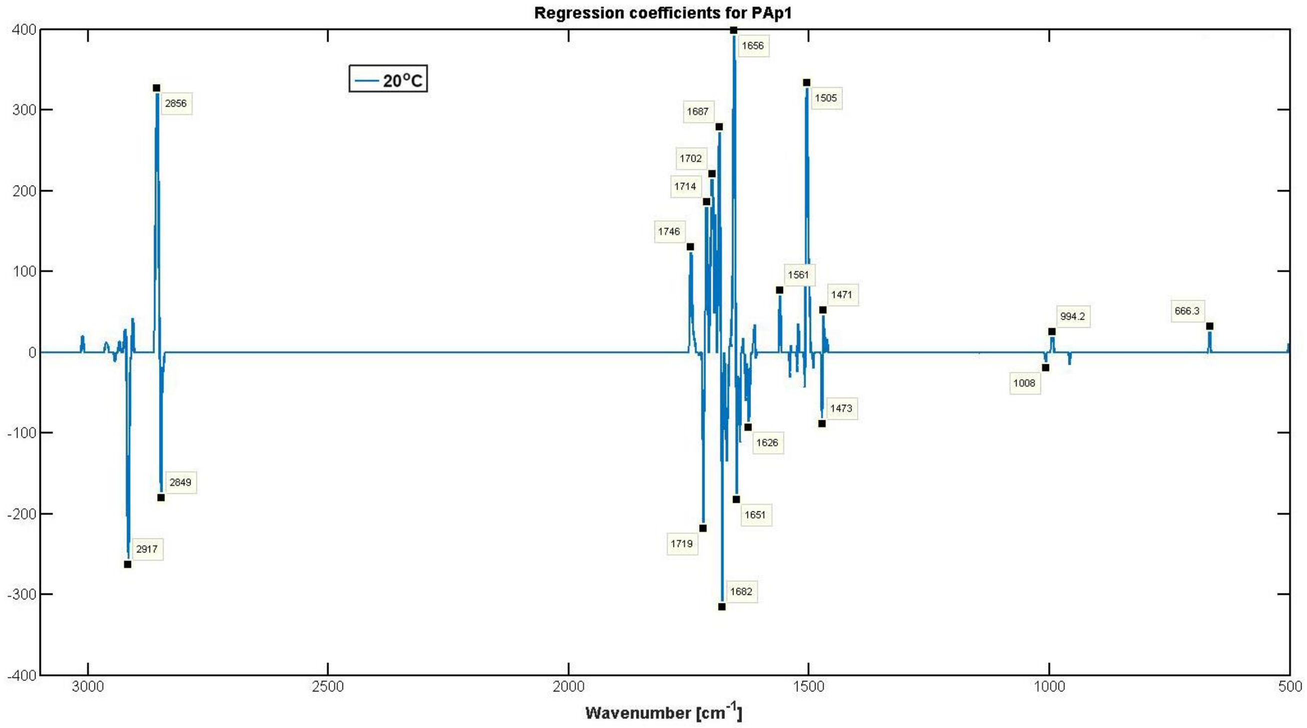

Fig. S11 (Part D) Classification based on growth conditions: The regression coefficient for *Poa alpina*, Sweden, 20°C; The regression coefficient for 14°C is inverse.

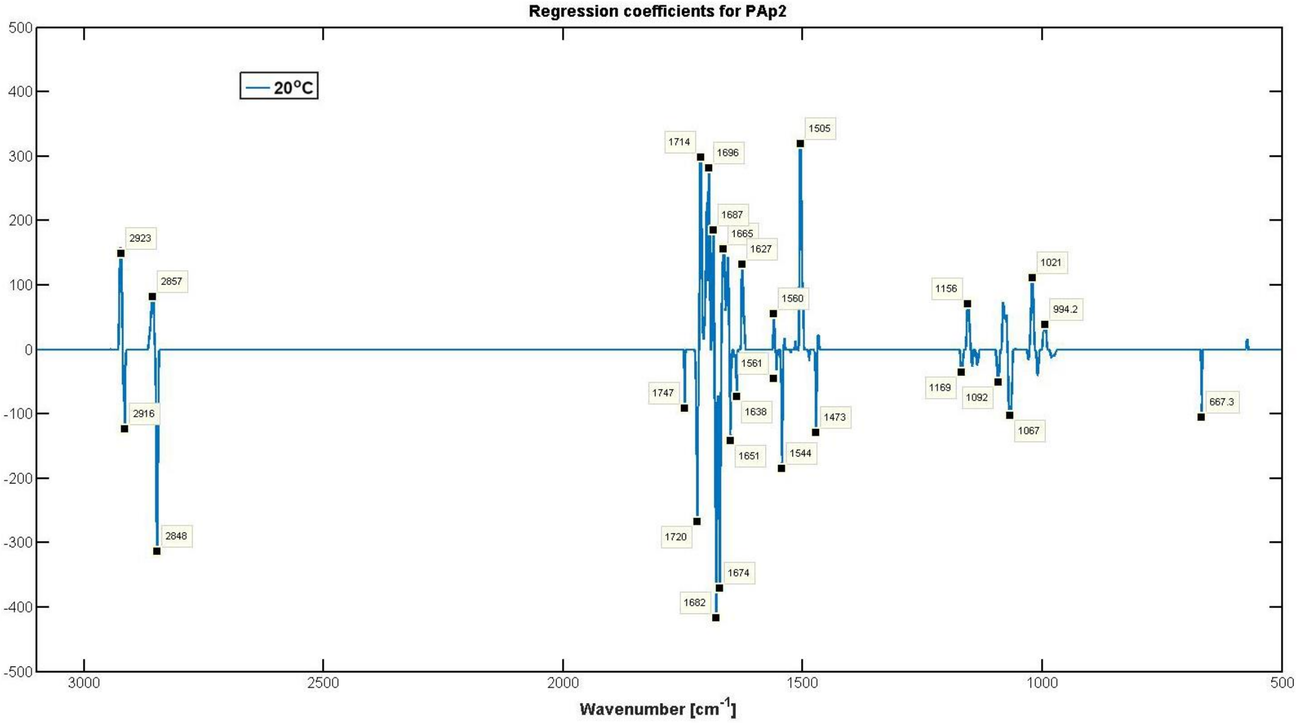

Fig. S11 (Part E) Classification based on growth conditions: The regression coefficient for *Poa alpina*, Italy, 20°C; The regression coefficient for 14°C is inverse.

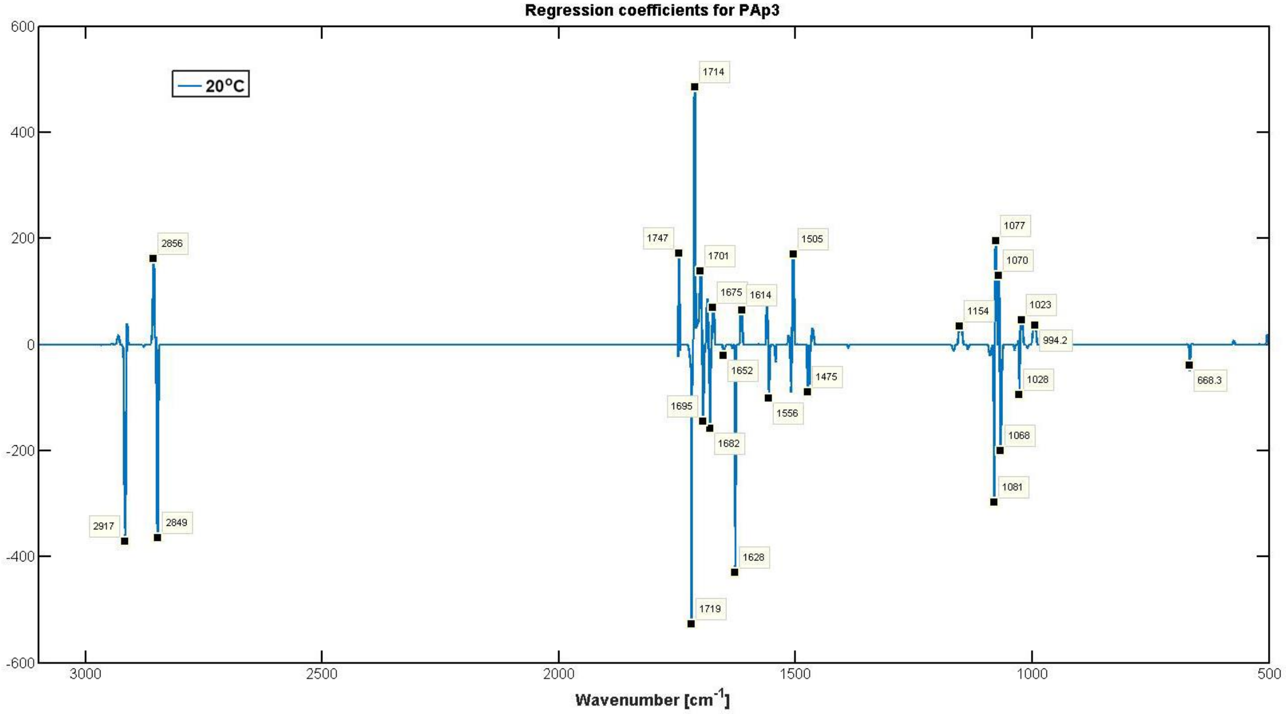

Fig. S11 (Part F) Classification based on growth conditions: The regression coefficient for *Poa alpina*, Norway, 20°C; The regression coefficient for 14°C is inverse.

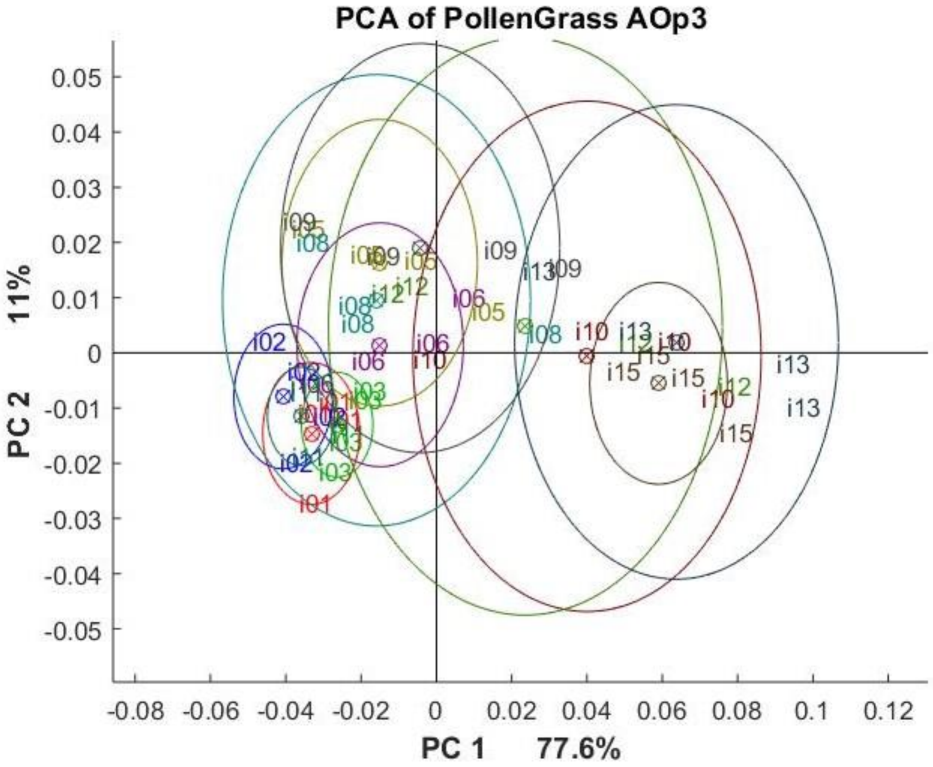

Fig. S12 (Part A) Principal component analysis for *Anthoxanthum odoratum*, Finland, showing clustering based on genotype.

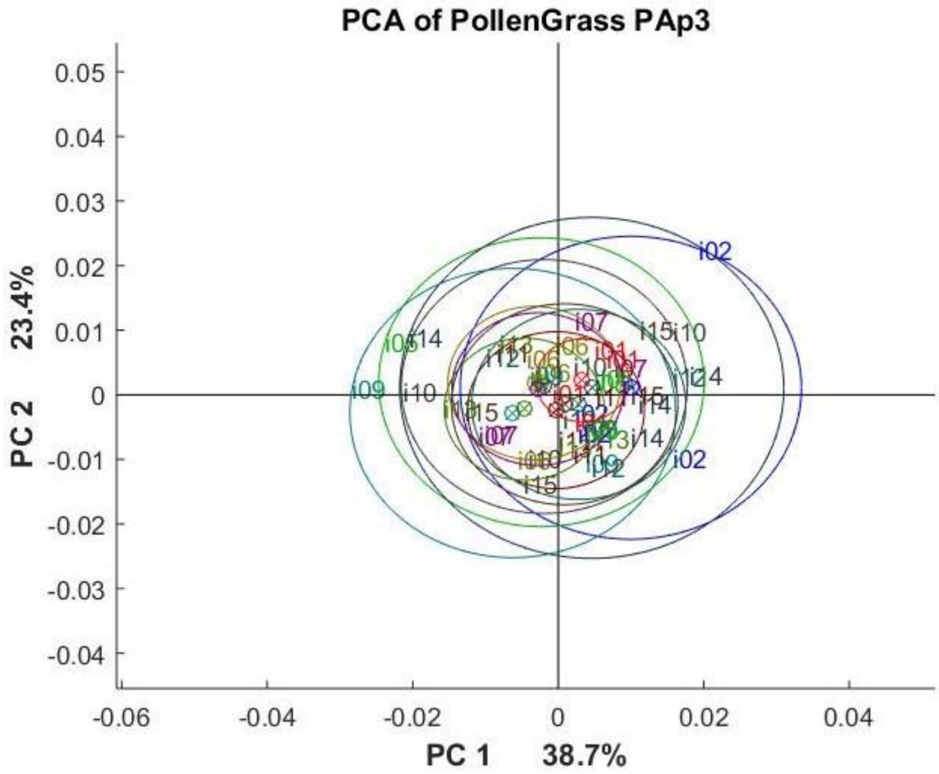

Fig. S12 (Part B) Principal component analysis for *Poa alpina*, Norway, showing clustering based on genotype.
